# Effectiveness of an intervention for reducing sitting time and improving health in office workers: three arm cluster randomised controlled trial

**DOI:** 10.1136/bmj-2021-069288

**Published:** 2022-08-17

**Authors:** Charlotte L Edwardson, Stuart J H Biddle, Stacy A Clemes, Melanie J Davies, David W Dunstan, Helen Eborall, Malcolm H Granat, Laura J Gray, Genevieve N Healy, Nishal Bhupendra Jaicim, Sarah Lawton, Benjamin D Maylor, Fehmidah Munir, Gerry Richardson, Thomas Yates, Alexandra M Clarke-Cornwell

**Affiliations:** 1 Diabetes Research Centre, University of Leicester, Leicester, LE5 4PW, UK; 2 NIHR Leicester Biomedical Research Centre, Leicester, UK; 3 Centre for Health Research, University of Southern Queensland, Springfield Central, QLD, Australia; 4 School of Sport, Exercise and Health Sciences, Loughborough University, Leicester, UK; 5 Leicester Diabetes Centre, University Hospitals of Leicester, Leicester, UK; 6 Baker Heart and Diabetes Institute, Melbourne, VIC, Australia; 7 Mary MacKillop Institute for Health Research, The Australian Catholic University, Melbourne, VIC, Australia; 8 Department of Health Sciences, University of Leicester, Leicester, UK; 9 Deanery of Molecular, Genetic and Population Health Sciences, The University of Edinburgh, UK; 10 School of Health and Society, University of Salford, Salford, Greater Manchester, UK; 11 School of Human Movement and Nutrition Sciences, The University of Queensland, Brisbane, QLD, Australia; 12 The Leicester Clinical Trials Unit, University of Leicester, Leicester, UK; 13 Centre for Health Economics, University of York, York, UK

## Abstract

**Objectives:**

To evaluate the effectiveness of an intervention, with and without a height adjustable desk, on daily sitting time, and to investigate the relative effectiveness of the two interventions, and the effectiveness of both interventions on physical behaviours and physical, biochemical, psychological, and work related health and performance outcomes.

**Design:**

Cluster three arm randomised controlled trial with follow-up at three and 12 months.

**Setting:**

Local government councils in Leicester, Liverpool, and Greater Manchester, UK.

**Participants:**

78 clusters including 756 desk based employees in defined offices, departments, or teams from two councils in Leicester, three in Greater Manchester, and one in Liverpool.

**Interventions:**

Clusters were randomised to one of three conditions: the SMART Work and Life (SWAL) intervention, the SWAL intervention with a height adjustable desk (SWAL plus desk), or control (usual practice).

**Main outcomes measures:**

The primary outcome measure was daily sitting time, assessed by accelerometry, at 12 month follow-up. Secondary outcomes were accelerometer assessed sitting, prolonged sitting, standing and stepping time, and physical activity calculated over any valid day, work hours, workdays, and non-workdays, self-reported lifestyle behaviours, musculoskeletal problems, cardiometabolic health markers, work related health and performance, fatigue, and psychological measures.

**Results:**

Mean age of participants was 44.7 years, 72.4% (n=547) were women, and 74.9% (n=566) were white. Daily sitting time at 12 months was significantly lower in the intervention groups (SWAL −22.2 min/day, 95% confidence interval −38.8 to −5.7 min/day, P=0.003; SWAL plus desk −63.7 min/day, −80.1 to −47.4 min/day, P<0.001) compared with the control group. The SWAL plus desk intervention was found to be more effective than SWAL at changing sitting time (−41.7 min/day, −56.3 to −27.0 min/day, P<0.001). Favourable differences in sitting and prolonged sitting time at three and 12 month follow-ups for both intervention groups and for standing time for the SWAL plus desk group were observed during work hours and on workdays. Both intervention groups were associated with small improvements in stress, wellbeing, and vigour, and the SWAL plus desk group was associated with improvements in pain in the lower extremity, social norms for sitting and standing at work, and support.

**Conclusions:**

Both SWAL and SWAL plus desk were associated with a reduction in sitting time, although the addition of a height adjustable desk was found to be threefold more effective.

**Trial registration:**

ISRCTN Registry ISRCTN11618007.

**Figure:**
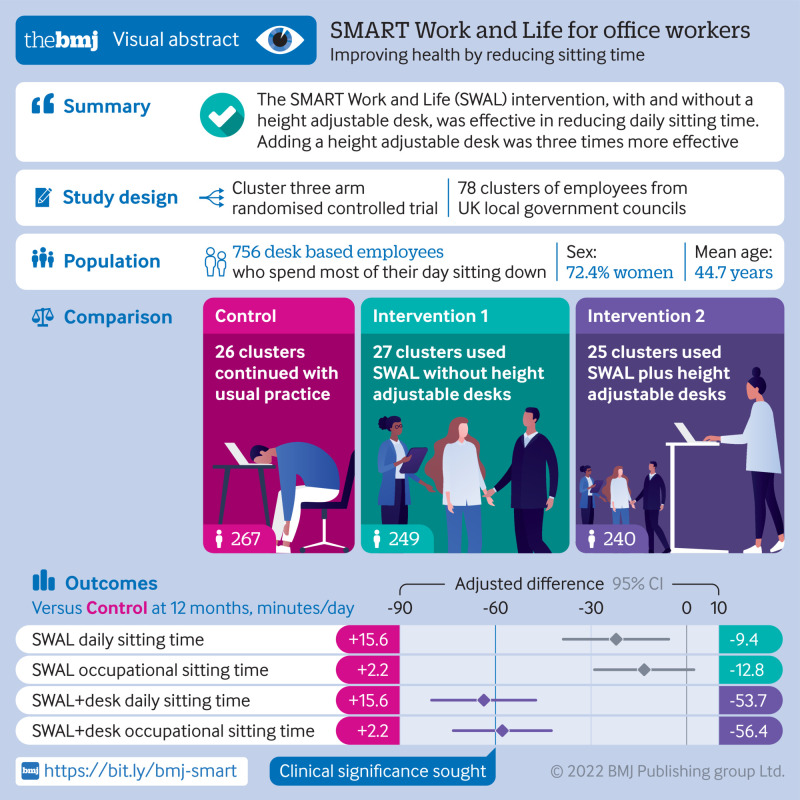


## Introduction

Data gathered from accelerometer based devices show that adults who are ambulatory spend about 9-10 hours of their day (60%) being sedentary (ie, sitting during waking hours).^1^ ^2^ ^3^ Office based workers are one of the most sedentary populations, spending 73% of their workday and 66% of their waking day sitting.^4^ This is of concern given the rapidly accumulating evidence that a greater amount of time spent sedentary is associated with: higher all cause and cardiovascular disease mortality rates^5^ ^6^ ^7^ ^8^, a higher risk of type 2 diabetes,^6^ ^7^ ^9^ incident cardiovascular disease,^6^ ^7^ ^10^ incident endometrial, colon, and lung cancers,^7^ ^11^ ^12^ anxiety,^13^ ^14^ depression,^15^ ^16^ ^17^ and a lower quality of life.^17^ ^18^ Together, this evidence highlights the potential health impact of sedentary behaviour on populations; and, as such, physical activity position statements and many national guidelines now include recommendations for reducing or regularly breaking up sedentary time,^19^ ^20^ ^21^ ^22^ including for the first time guidelines from the World Health Organization.^23^

With office workers exhibiting high levels of sedentary behaviour, interventions that focus on reducing sitting time in the workplace have emerged.^24^ ^25^ Although these interventions have shown promising results, particularly those involving height adjustable desks, the quality of the evaluation of these interventions was deemed to be very low to low owing to a lack of non-biased cluster randomised controlled trials, small sample sizes (most studies included 20-50 participants), a lack of longer term follow-up, and lack of valid and reliable assessments of sedentary behaviour.^24^ These limitations highlight the need for larger cluster randomised controlled trials with long term follow-up. Two recent large randomised controlled trials evaluated multicomponent interventions to reduce sedentary behaviour involving a height adjustable desk and observed differences of 45 minutes per eight hour workday in sitting time in favour of the intervention compared with control at 12 month follow-up^26^ ^27^; however, both of these randomised controlled trials focused primarily on reducing sitting time at work and showed no impact on behaviour outside of work. Evidence suggests that office workers also tend to be highly sedentary outside of work,^28^ and therefore by extending workplace interventions to deal with sedentary behaviour both during work and at other times might potentially achieve greater impacts on health. Furthermore, multicomponent interventions have been delivered alongside the provision of height adjustable desks, and as such have prevented understanding of whether behaviour change can be achieved without this environmental change, and how much extra benefit can be achieved by the environmental change. This is important knowledge for organisations that invest in workplace wellbeing programmes for employees, as the provision of height adjustable desks involves cost.

To address these research gaps in evaluation methods and intervention design we have built on our previous multicomponent intervention, Stand More AT Work (SMArT Work), which was shown to successfully reduce occupational sitting time over 12 months.^26^ Based on the results of the SMArT Work randomised controlled trial, process evaluation, and stakeholder input, we created the SMART Work and Life (SWAL) intervention.^29^ This intervention focuses on a whole day approach to reducing sitting time and is designed for implementation by trained workplace champions within the target organisation: this is an important step forward compared with previous trials, as the study allows for evaluation of the real world implementation of the intervention. To evaluate the effectiveness of the SWAL intervention (with and without a height adjustable desk), we conducted a large multisite cluster randomised controlled trial in a sample of desk based employees from local government, one of the largest employers in the United Kingdom. The primary objective was to establish whether SWAL, delivered with and without a height adjustable desk and by workplace champions, was associated with changes in daily sitting time (ie, during and outside work) compared with usual practice (control group) at 12 month follow-up. If both interventions were shown to be effective compared with the control group, a secondary objective was to determine if one intervention was more effective than the other. To contribute to the evidence base on the extent to which sedentary behaviour interventions impact on health, wellbeing, and work related outcomes, secondary outcomes included physical activity, physical health, mental health, and work related health and performance.

## Methods

### Study design

This three arm cluster randomised controlled trial, with follow-up at 3, 12, and 24 months, is reported according to the Consolidated Standards of Reporting Trials (CONSORT) statement for cluster randomised controlled trials,^30^ and the CONSORT 2021 statement for trials that were modified because of the covid-19 pandemic.^31^ The trial protocol has been published^29^ (see supplementary table 1 for changes to the protocol during the study). Clusters, comprising participants from defined offices, departments, or teams, were randomised to one of three interventions: SWAL without a height adjustable desk, SWAL with a height adjustable desk, and usual practice (control). Randomisation was stratified by council area (Leicester, Liverpool, and Greater Manchester) and cluster size (<10 and ≥10 participants). The advent of the covid-19 pandemic and the subsequent lockdown in March 2020 in the UK necessitated changes from the published protocol.^29^ As a result of these extenuating circumstances, the trial steering committee and the funder (National Institute of Health and Care Research) recommended removal of the 24 month follow-up, with data for the primary outcome to be collected at the 12 month follow-up and secondary outcomes to be reported at three and 12 months. All 12 month data had been collected by February 2020 and the 24 month data collection had not yet started.

### Setting, clusters, and participants

Six councils agreed to take part: two in Leicester, one in Liverpool, and three in Greater Manchester. We tailored the recruitment methods to each council, but in all councils the study was advertised through the staff intranet, with some councils also displaying posters and including information in newsletters. In three councils, participants were also invited to attend a 45 minute presentation about the study. Participants were eligible to take part in the study if they were employed by the council and were aged ≥18 years, spent most of their day sitting (self-report to question “Roughly, how much of your day do you spend sitting?”), worked at least 60% full time equivalent, could give informed consent, and were able to walk unassisted. We excluded those who were pregnant, already used a height adjustable desk, were unable to provide informed consent, or were unable to communicate in English. Clusters comprised people in a shared office space, which could be made up of different teams and departments, or members of the same team but working in different office spaces.

Recruitment took place between February 2018 and January 2019. Baseline data were collected between May 2018 and February 2019, three month data between September 2018 and June 2019, and 12 month data between June 2019 and February 2020. All participants provided informed consent on entering the study.

### Randomisation arms

#### Intervention groups

We adapted the SWAL intervention from our previous intervention, SMArT Work.^32^ The SWAL intervention is grounded in social cognitive theory,^33^ organisational development theory,^34^ habit theory,^35^ self-regulation theory,^36^ and relapse prevention theory.^37^ The intervention includes a range of multifaceted strategies (organisational, environmental, individual, and group), which draw upon the principles of the behaviour change wheel and the associated COM-B (capability, opportunity, motivation, and behaviour) approach.^38^

One intervention group received SWAL only and the other group received SWAL and a height adjustable desk.

*Organisational strategies—*Support of senior leaders was secured through a series of business case documents and videos, which articulated the importance of reducing employee sitting behaviours, the positive impact this reduction could have on workplace culture, and how reduction in sitting time might be achieved without disrupting performance and productivity. Workplace champions, who were council employees enrolled as participants in the study, were identified within each cluster and facilitated the interventions. Workplace champions attended a training session (three hours) delivered by a behaviour change education team to equip them with the skills and knowledge for facilitation of the interventions. The senior management team within each council allowed workplace champions protected time each month for facilitation of the interventions.

*Environmental strategies*—The intervention promoted small scale restructuring of the office environment (eg, relocation of printers and wastepaper bins, standing meetings, standing areas in shared space) to encourage more frequent movement around the office. Participants were also encouraged to think about their home environment. Motivational posters were embedded in the office environment. The workplace champions demonstrated positive examples within the working environment (ie, behavioural modelling).

Clusters randomised to the SWAL plus desk group received a height adjustable desk to encourage them to transition between sitting and standing postures while working. Participants were able to select their preferred desk type and colour (black or white) from four models: Deskrite 100 (Posturite, Berwick, UK), Yo-Yo Desk Mini, Yo-Yo Desk 90, or Yo-Yo Desk Go (Sit-Stand Trading, Swindon, UK). All the desks were designed to sit on top of the participants’ existing workstation. Participants were provided with an information booklet and guidance from the research team on how to use the desk appropriately when in the sitting and standing positions, as well as recommendations based on the sedentary office expert statement^39^ on how much standing to achieve throughout the day (ie, gradually work towards two hours of standing and light movement and eventually towards four hours). The importance of avoiding prolonged standing was also highlighted.

*Group and individual strategies*—Workplace champions provided participants with a link to a one-off online education session that covered the adverse health consequences of excessive sitting and reinforced the benefits of breaking up sitting time and reducing overall sitting time. The session also encouraged participants to estimate their own sitting time at work and at home and to think about strategies to reduce and break up sitting time in both environments; provided a range of ideas on how to reduce and break up sitting time at work and at home; covered identification of barriers, and goal setting; provided information on the importance of self-monitoring and prompts for behaviour change; and suggested a range of free smartphone enabled applications and computer software or extensions to use at work and home. Participants were provided with a range of downloadable resources, including posters, ideas to reduce and break up sitting, and an action plan and goal setting sheet. The main message of the intervention was to sit less (<50% of the waking day spent sitting)^40^ and move more often (every 30 minutes).^41^ Sitting less and moving more challenges were provided that could be completed individually, in groups, or with family and friends, with the suggestion to take part in these challenges three times during the 12 month study period. Group catch-up sessions were encouraged at three and nine months to provide an opportunity for participants to collectively review key messages of the intervention, brainstorm ideas, discuss any barriers and facilitators to reducing sitting time, and develop new goals and action plans.

The workplace champions were responsible for providing participants with a link to the online education session, sending out monthly emails, putting up posters, organising and facilitating sitting less challenges and group catch-up sessions, and acting as positive role models. Workplace champions were not provided with any financial incentive to facilitate the intervention; however, they were provided with a £20 voucher for completing evaluation documentation, which detailed when they delivered the intervention activities (information used for the process evaluation).

#### Control group

The control group carried on with usual practice for the length of the study.

### Participant characteristics

Data were collected on participants’ date of birth, sex, ethnicity, marital status, education level (reported highest level of qualification), smoking status (current, former, never), household composition (number of children <18 years), postcode, medical history (answering yes or no to a list of 15 health related conditions, such as type 2 diabetes, heart disease, high blood pressure), drugs (answering yes or no to use of common drugs for reducing lipid levels and blood pressure), job role, pay grade, site of work, contracted weekly working hours, length of employment at the council (years and months), and number of people in the office and department. Data from the activPAL device were used to describe the percentage of time participants spent sitting (total and prolonged), standing, and stepping; number of steps; moderate to vigorous activity stepping time; number of sit to upright transitions; valid waking time of the activPAL; and number of valid activPAL days during work and daily hours.

### Main outcome measures

The primary outcome was daily (across all waking hours) sitting time at the 12 month follow-up, measured using the activPAL3 micro accelerometer (PAL Technologies, Glasgow, UK), which can distinguish between sitting or lying, static standing, stepping time, and transitions between sitting and standing.^42^ Participants were asked to wear the device for 24 hours a day for eight days. The activPAL device was initialised to record at a sampling frequency of 20 Hz: the device was waterproofed with a nitrile sleeve and applied (by the participant) to the midline anterior aspect of the thigh using a transparent dressing. Participants were provided with a diary to record the times they got into bed, went to sleep, woke up, and got out of bed while wearing the device, as well as indicating which days were workdays and which non-workdays, and the start and finish times of each workday. Participants were also asked to indicate whether each day was a typical day, and, if not, why, and to note any times that they removed the device and why.

The secondary outcomes, measured at three and 12 months, unless specified otherwise, were:

*Other sedentary behaviour, physical activity, and lifestyle variables*—Other variables of interest calculated from the activPAL data included daily sitting time at three months, prolonged sitting time (≥30 minutes), standing time, stepping time, time spent stepping at moderate or greater intensity physical activity (≥100 steps/min), number of steps, and number of sit to upright transitions. All these variables were calculated as daily time on any valid day, during work hours, and on workdays and non-workdays (splitting by workdays and non-workdays was not prespecified but is important for understanding when behaviour change occurs). In addition to the activPAL, participants were asked to wear an accelerometer on their non-dominant wrist (Axivity AX3; Axivity, Newcastle, UK) for 24 hours a day for eight days. These devices were initialised with a sampling frequency of 100 Hz and a dynamic range of ±8 *g* (where *g* is equal to the Earth’s gravitational pull). Variables calculated included time spent in light (40-100 milligravity (m*g*)) and moderate to vigorous physical activity (>100 m*g* (where 80% of a 60 second window exceeded 100 m*g*)),^43^ sleep duration, and sleep efficiency.

*Self-reported lifestyle behaviours—*self-reported percentage of time spent sitting, standing, and walking were assessed using an adapted version of the Occupational Sitting and Physical Activity Questionnaire, which asks participants to report sitting, standing, and walking percentage rather than, as in the original version, sitting, standing, walking, and heavy labour.^44^ Participants were also asked to estimate the hours that they spent sitting, and the number of times each hour they broke up sitting during the workday.^45^ The Past Recall of Sedentary Time was used to assess time spent sitting in different contexts outside of working hours.^46^ Percentage of time participants spent in the office and at their desk during the workday was reported, as well as number of work days and hours worked each week. Average consumption of snacks, soft drinks, fruit and vegetables, and alcohol was assessed using questions from the Whitehall II study.^47^ Self-reported sleep duration and quality were captured using the Pittsburgh Sleep Quality Index.^48^

*Physical health—*Height was measured to the nearest 0.1 cm, body weight to the nearest 0.1 kg, body fat percentage (MBF-6000 Bioimpedance Scales; Marsden, Rotherham, UK) to the nearest 0.1%, and waist circumference to the nearest 0.1 cm. After participants had rested for five minutes, three measurements of blood pressure (Omron, Henfield, UK) were taken, with the last two averaged and used in the analysis. Fasting (at least 10 hours) point of care testing included measures of glycated haemoglobin (Quo-Test HbA1c analyser; EKF Diagnostics, Cardiff, UK), cholesterol (high density lipoprotein, low density lipoprotein, and total), triglycerides, and blood glucose (CardioChek Plus; PTS Diagnostics, IN). A clustered cardiometabolic risk score (non-prespecified outcome) was created using waist circumference, triglyceride level, high density lipoprotein cholesterol level, systolic and diastolic blood pressure, and fasting glucose level.^49^ Musculoskeletal symptoms over the past three months and seven days were self-reported using the Standardised Nordic Questionnaire.^50^ The Fatigue Scale was used to assess both mental and physical fatigue.^51^

Participants in all three groups (intervention and control) received a copy of their anthropometric and health results from the baseline, three month, and 12 month visits, as well as a £10 gift voucher after each visit on return of complete data. These decisions were taken after input from patient and public involvement before the start of the study.

*Mental health*—The Hospital Anxiety and Depression Scale assessed anxiety and depression symptoms.^52^ Stress was reported using the Perceived Stress Scale.^53^ Emotion was assessed through the Positive and Negative Affect Schedule.^54^ The WHO-5 Wellbeing Index was used to measure psychological wellbeing.^55^ Health related quality of life (health state and visual analogue scale) was measured using the EQ5D-5L.^56^ ^57^ ^58^

*Work related health and performance*—Single item scales were used to assess perceived job performance^59^ and job satisfaction.^60^ The Utrecht Work Engagement Scale was used to measure work engagement overall and subscales of vigour, dedication, and absorption.^61^ Occupational fatigue was measured using the Need for Recovery Scale.^62^ The Work Limitations Questionnaire was used to measure sickness presenteeism overall and subscales of time management, physical demands, mental-interpersonal demands, and output demands.^63^ The Health and Safety Executive Management Standards Indicator Tool was used to assess perceived demands, control, and support in relation to workload.^64^ Self-reported sickness absence (number of episodes) over the past three months was reported at baseline and 12 month follow-up, and organisation records provided information on number of episodes and duration of absences in the 12 months before the study and the 12 month duration of the study.

*Social norms, cohesion, and support*—Organisational social norms (eg, my colleagues would not mind if I chose to stand up while working at my desk) around sitting and standing at work were assessed with eight items using a five point Likert scale.^65^ The Copenhagen Psychosocial Questionnaire-II captured the presence and extent of cohesion, cooperation, and community in workplace teams using three, six point Likert scale items.^66^ Participants were also asked about the support they received from their organisation, manager, colleagues, and family for sitting less and moving more often.^67^

*Adverse events—*During the study, information on adverse events was collected from the participants. An adverse event was defined as any untoward medical occurrence that did not necessarily have to be causally related to the study or intervention. Participants were asked to inform us of any adverse event by email or phone, or in person at the measurement visits.

### Accelerometer data processing

ActivPAL data were cleaned and processed using a freely available java application (University of Leicester, Leicester, UK, https://github.com/UOL-COLS/ProcessingPAL). This application enables the user to separate valid waking data from everything else (time in bed, prolonged non-wear of the device, and invalid data).^68^ We created heat maps of the processed data and visually checked for any occasions where the algorithm had potentially misclassified waking wear data, and vice versa. On any such occasion we compared the self-reported wake and sleep times with the processed data, and if these confirmed misclassification of data by two hours or more, we corrected the data. Self-reported logs were also checked for scenarios where data should be removed—for example, if participants removed the device for swimming or it was not a typical day. Once the data had been cleaned, we calculated summary variables on the valid waking wear data. We excluded the first day of data collection. A valid wear day for daily, workday, and non-workday data variables was defined as wearing the device for ≥10 hours daily, achieving ≥1000 steps daily, and spending <95% of the day in any one behaviour.^68^ To be included in the analysis of daily data we required participants to have at least one valid day (any day of the week was considered). Short (≤5 hours) and long (≥12 hours) wear times during working hours were checked against the self-reported logs. For data variables during working hours, a time period had to have ≥3.5 hours of data (≥50% of full time equivalent workday).

Axivity data files were processed through R package GGIR version 1.9-0 (http://cran.r-project.org),^69^ using R version 4.0.2. To generate outcome variables based on a complete 24 hour cycle, we used the default non-wear setting in GGIR. Briefly, we replaced invalid data with mean acceleration values for similar time points from different days for each participant.^70^ A valid day of daily data was defined as detection of wearing the device >16 hours within a 24 hour window, or when wear was detected for each 15 minute period over a 24 hour cycle.^70^ We excluded the first day of wear. Sleep metrics were derived using an estimated sleep period time window based on sustained bouts of inactivity; estimated arm angles were averaged over five second epochs and treated as sustained inactivity or potential sleep periods if the angle change was less than 5° over a rolling five minute window.^71^ We excluded data for the first and last night because the recording period started and ended at midnight. Visual reports were generated and compared for accuracy against participant wake and sleep time diaries. Obvious inaccuracies in the predicted sleep window based on viewing the data resulted in the removal of the window altogether.^71^ The same number of valid days and work hours criteria were applied to the Axivity data that were applied to the activPAL data.

### Statistical analysis

#### Sample size

We determined that a sample size of 420 participants and 10 clusters in each arm would provide more than 90% power to detect a 60 minute difference in overall sitting time using multilevel models with a two tailed significance level of 5%. This calculation assumed a standard deviation of 90 minutes, an intraclass correlation coefficient of 0.05, a coefficient of variation of 0.54 (cluster range 15-45), and an average cluster size of 20, allowing for multiple comparisons with the control group. We inflated the number of clusters in each arm by 1 to allow for whole cluster drop-out, and the number of participants was inflated by 30% to allow for potential individual loss to follow-up and non-compliance with activPAL, giving a total sample size of 660 participants to be recruited, with 11 clusters in each arm.

During the recruitment process it became clear that the observed average and variability of cluster size were different to those assumed in the original sample size calculation in the published protocol.^29^ With the agreement of the data monitoring and ethics committee, the average cluster size was changed from 20 to 10 and the variability in cluster size from 0.54 to 1.42 (cluster size range 4-38), and the inflation for loss to follow up and non-compliance with wearing the activPAL device was increased from 30% to 40%. This resulted in 690 participants from 72 clusters needed to provide more than 90% power for the primary outcome. Sample size calculations were performed using Stata.

#### Data analysis

Baseline summary statistics were summarised by randomisation group. Those participants with primary outcome data at baseline and 12 months (included in the primary analysis) were compared with those without such data. The primary outcome, daily sitting time at 12 months on any valid day (minimum one day), was analysed on a complete case basis using a linear multilevel model. Sitting time at the 12 month follow-up was included as the outcome, adjusting for daily sitting time at baseline and average valid activPAL waking wear time across baseline and 12 month follow-up. The model also included a categorical variable for randomisation group (control as reference), and terms for the stratification factors (area: Leicester, Liverpool, and Greater Manchester, and cluster category size). Office clusters were included as a random effect. If both intervention arms were shown to be effective, a secondary exploratory analysis was planned to evaluate if one intervention was more effective than the other.

#### Sensitivity analyses

Several sensitivity analyses were conducted on daily sitting time at 12 months and one key secondary outcome (sitting during work hours at 12 months): intention to treat, per protocol, standardising activPAL waking and work hours, and the effect of a different number and type of valid activPAL days:

*Intention-to-treat analysis*—We performed intention-to-treat analysis with missing data imputed using multilevel multiple imputation, taking account of clustering. The model imputed missing values for daily sitting time at baseline, three months, and 12 months, body mass index (BMI) at baseline and three months, and average activPAL waking wear time across baseline and 12 months. To inform the imputation, the model included the non-missing covariates of sex, ethnicity, age, cluster size category, and area. The multilevel multiple imputation used 20 imputations, 10 000 burn-in iterations, and 10 000 between imputation iterations, carried out separately by randomisation arm. We fitted the same model as specified for the primary analysis to each of the 20 imputed datasets and combined the data using Rubin’s rules to estimate the intervention effect.

*Per protocol analysis*—In the per protocol analysis, we excluded participants who did not provide valid activPAL data at baseline and 12 month follow-up, control participants who reported having access to a height adjustable desk, participants who were seen plus or minus two months outside of their expected follow-up date, participants who spent <50% of their day sitting at baseline, and participant clusters who had no workplace champion assigned or the workplace champion had dropped out within the first three months of the intervention.

*Standardising activPAL waking and work hours*—activPAL data were normalised to a 16 hour waking day and an eight hour workday.^27^ ^72^

*Effect of different number and type of valid activPAL days*—The effect of the minimum number of valid activPAL days and type of days (ie, any valid days and workdays) was assessed.

To assess if the intervention effect was different between randomisation groups, we conducted several subgroup analyses: area (Leicester, Liverpool, Greater Manchester), cluster size category (small ≤10; large >10), sex (men, women), age (below or above the median),^26^ and BMI (normal, overweight, or obese (≥25)),^73^ and worker status (part time, full time). We included an interaction term between intervention arm and subgroup to assess the level of heterogeneity in intervention effect between the subgroups. An estimate of the intervention effect (ie, difference between subgroups) and 95% confidence interval are presented for each subgroup alongside the P value for the interaction term. In response to reviewer’s comments, we repeated the subgroup analysis for age and BMI as continuous variables within the model to assess if the intervention effect changed as these variables increased.

Using similar methodology to the primary outcome, we analysed key secondary activPAL assessed outcomes (measured at three and 12 months unless specified otherwise): sitting time (at three months), prolonged sitting time, standing time, and stepping time, calculated daily during work hours and on workdays and non-workdays. No corrections for multiple testing were made, and P values and 95% confidence intervals are presented for these variables only.

For all other secondary outcomes, only descriptive analyses with no statistical testing were performed at three and 12 months: continuous data that were approximately normally distributed were summarised as means and standard deviations, and skewed data with medians and interquartile ranges. Ordinal and categorical data were summarised using frequency counts and percentages.

The analysis was performed using Stata (version 16.0). Multilevel multiple imputation was implemented through REALCOM-IMPUTE software in conjunction with Stata or using the *jomo* package in R (Studio version 1.3.959). All tests and reported P values were two sided. Estimates are presented with 95% confidence intervals, with the exception of the primary analysis of the primary outcome (daily sitting time), which are presented with 97.5% confidence intervals.

### Patient and public involvement

Office workers, workplace champions, and managers within the target organisation were involved in the study design during the grant application process and the study delivery phase. During the grant application phase, the purpose and design of the study as well as the suggested intervention strategies were presented to two large groups of council employees: as a result of these meetings, the study design included using finger prick blood testing rather than taking venous blood samples, participants receiving feedback on health measures, and incentives for attending follow-up. During the study set-up and delivery, a council employee advisory group met several times and provided advice on delivery of the interventions (feedback showed that workplace champions would not be comfortable delivering the initial education session because of the training and planning time required, so this session was delivered online instead), recruitment processes (feedback was provided on participant documents and recruitment messages and strategies within the council), installation of the height adjustable desk, and troubleshooting. Two council employees were also part of the trial steering committee, which met twice a year during the study.

## Results

**Fig 1:**
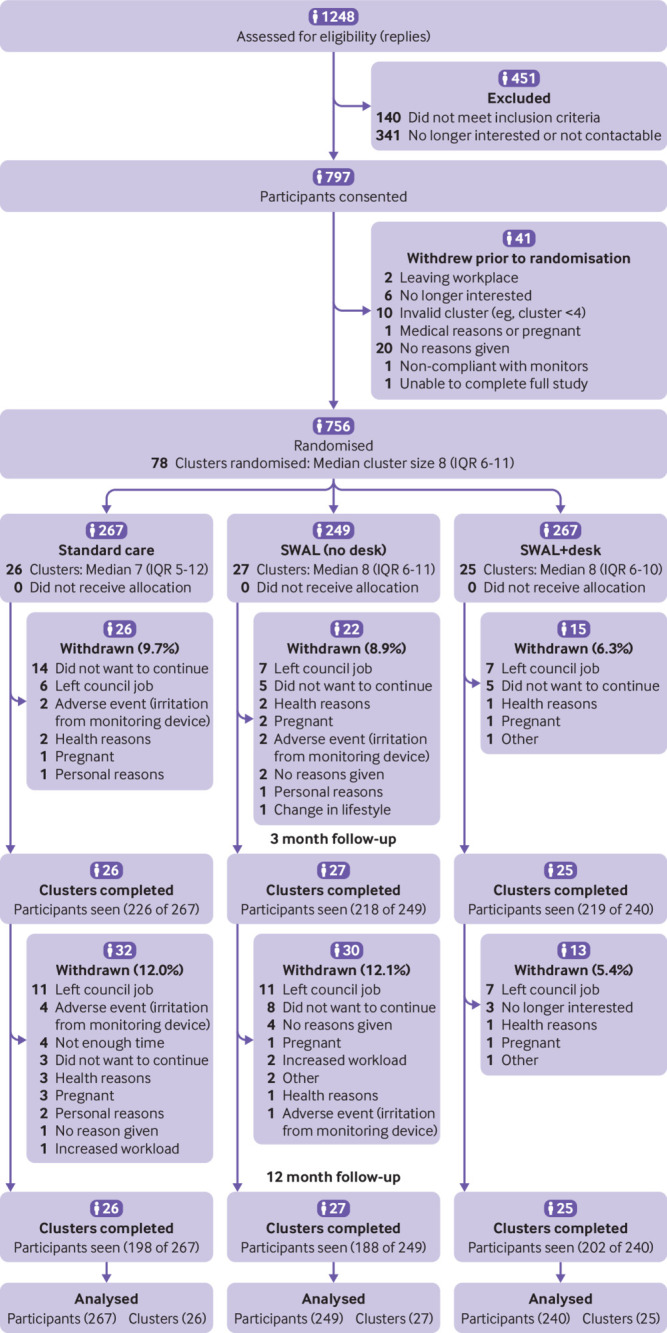
Flow of participants through study

Figure 1 shows the flow of clusters and participants through the study. Overall, six councils in three areas of England (Leicester, Liverpool, Greater Manchester) were recruited, from which 756 participants across 78 clusters were randomised: 26 office clusters to the control arm (267 participants), 27 clusters to the SWAL arm (249 participants), and 25 clusters to the SWAL plus desk arm (240 participants). No council or whole cluster drop-out occurred during the study; however, 12.3% of participants at three months (n=93) and 22.2% (n=168) of participants at 12 months did not attend follow-up measurements. Fewer participants dropped out from the SWAL plus desk arm than the SWAL intervention and control arms (fig 1).

### Baseline characteristics

Table 1 presents the characteristics of the office clusters and the individual participants within these clusters at baseline. Median cluster size was 8 (interquartile range 6-11). The mean age of participants was 44.7 (SD 10.5) years, 72.4% (n=547) were women, 74.9% (n=566) were white British, and the mean BMI was 26.5 (SD 5.9). Most participants (85.0%) worked full time. No significant differences were found between those with available primary outcome data at both baseline and 12 months and those without for the characteristics reported in table 1, except for age (those who were older were more likely to have available data; 41.6 *v* 45.8 years, P<0.001).

**Table 1:** Baseline characteristics of office clusters and participants randomised to the SMART Work and Life (SWAL) intervention with or without a desk or to usual practice (control). Values are means (standard deviations) unless stated otherwise

Characteristics	Control	SWAL	SWAL+desk	Total
**Office clusters**				
No of clusters	26	27	25	78
Cluster size (No (%)):				
Small (<10)	17 (65.4)	17 (63.0)	16 (64.0)	50 (64.1)
Large (≥10)	9 (34.6)	10 (37.0)	9 (36.0)	28 (35.9)
Median (IQR) cluster size	7 (5-12)	8 (6-11)	8 (6-10)	8 (6-11)
No (%) of clusters by area:				
Leicester	14 (53.9)	15 (55.6)	13 (52.0)	42 (53.9)
Liverpool	4 (15.4)	5 (18.5)	6 (24.0)	15 (19.2)
Greater Manchester	8 (30.8)	7 (25.9)	6 (24.0)	21 (26.9)
**Participants**				
No of participants	267	249	240	756
Cluster size (No (%)):				
Small (<10)	106 (39.7)	108 (43.4)	104 (43.3)	318 (42.1)
Large (≥10)	161 (60.3)	141 (56.6)	136 (56.7)	438 (57.9)
No (%) of participants by area:				
Leicester	179 (67.0)	141 (56.6)	137 (57.1)	457 (60.4)
Liverpool	22 (8.2)	35 (14.1)	44 (18.3)	101 (13.4)
Greater Manchester	66 (24.7)	73 (29.3)	59 (24.6)	198 (26.2)
**Personal characteristics**				
Age (years)	44.5 (11.2)	43.8 (9.93)	45.9 (10.1)	44.7 (10.5)
Sex:				
No (%) men	71 (26.6)	64 (25.7)	74 (30.8)	209 (27.6)
No (%) women	196 (73.4)	185 (74.3)	166 (69.2)	547 (72.4)
Ethnicity (No (%)):				
White British	192 (71.8)	187 (75.1)	187 (78.0)	566 (74.9)
Asian	58 (21.6)	49 (19.7)	41 (17.1)	148 (19.6)
Other	17 (6.2)	13 (5.2)	12 (5.0)	42 (5.6)
No (%) with degree level or higher	152 (57.1)	170 (68.3)	134 (55.8)	456 (60.5)
No (%) married or cohabiting	189 (71.0)	178 (71.4)	183 (76.2)	550 (72.8)
No (%) current smoker (No (%))	10 (3.8)	15 (6.0)	9 (3.8)	34 (4.5)
No of people in household	2.94 (1.27)	2.96 (1.33)	2.96 (1.25)	2.95 (1.3)
No of children in household	0.64 (0.97)	0.73 (0.96)	0.63 (0.93)	0.67 (1.0)
No (%) full time worker (≥35 h/wk)	229 (85.8)	205 (83.0)	206 (86.2)	640 (85.0)
No (%) staff manager or supervisor	85 (32.0)	87 (35.4)	90 (37.5)	262 (34.8)
Duration of employ at council (years)	12.6 (9.87)	11.6 (8.78)	13.1 (9.59)	12.4 (9.4)
Duration in current role (years)	5.50 (6.35)	5.34 (4.59)	5.48 (4.89)	5.44 (5.4)
Contracted weekly hours	35.3 (3.65)	35.3 (3.60)	35.4 (3.48)	35.4 (3.6)
No of people in office	68.7 (71.5)	61.2 (66.2)	47.2 (36.6)	59.4 (61.1)
Biometric measurements:				
Weight (kg)	71.6 (17.1)	75.1 (18.1)	73.8 (17.6)	73.4 (17.6)
Body mass index	25.8 (5.60)	27.3 (6.42)	26.4 (5.68)	26.5 (5.9)
Per cent body fat	32.4 (9.26)	33.7 (9.44)	32.3 (9.27)	32.8 (9.3)
Waist circumference (cm)	86.6 (13.7)	89.0 (15.0)	89.2 (14.4)	88.2 (14.4)
Systolic blood pressure (mm Hg)	116.9 (14.5)	119.0 (17.3)	119.2 (16.6)	118.3 (16.2)
Diastolic blood pressure (mm Hg)	78.1 (9.46)	79.4 (10.7)	79.9 (11.1)	79.1 (10.4)
Median (IQR) fasting glucose (mmol/L)	5.30 (4.90-5.70)	5.40 (5.00-5.80)	5.40 (5.00-5.80)	5.30 (5.00-5.75)
Median (IQR) HbA1c (mmol/L)	32.7 (30.5-35.1)	33.3 (31.3-35.6)	33.9 (31.1-36.2)	33.3 (30.9-35.7)
Median (IQR) HbA1c (%)	5.14 (4.94-5.36)	5.20 (5.01-5.41)	5.25 (5.00-5.46)	5.20 (4.98-5.42)
Median (IQR) triglycerides (mmol/L)	1.04 (0.80-1.38)	1.05 (0.83-1.38)	1.05 (0.82-1.41)	1.05 (0.82-1.39)
Cholesterol (mmol/L):				
High density lipoprotein	1.46 (0.38)	1.41 (0.42)	1.42 (0.39)	1.43 (0.40)
Low density lipoprotein	2.52 (0.96)	2.65 (1.26)	2.56 (1.00)	2.58 (1.08)
Total	4.64 (1.04)	4.71 (1.07)	4.67 (1.06)	4.67 (1.06)
**activPAL variables**				
Daily values:				
Sitting (min)	601.6 (83.7)	605.2 (84.3)	609.4 (78.5)	605.2 (82.2)
Prolonged (≥30 min) sitting (min)	316.6 (100.2)	313.8 (97.6)	324.2 (102.7)	318.1 (100.1)
Standing (min)	230.8 (66.5)	226.4 (70.5)	231.9 (70.1)	229.7 (68.9)
Stepping (min)	109.3 (33.5)	108.7 (31.3)	109.2 (33.3)	109.1 (32.7)
No of steps	9291.0 (3209.1)	9286.4 (3121.3)	9230.9 (3228.7)	9270.5 (3182.6)
Median (IQR) MVPA stepping time (min)	23.3 (14.0-36.4)	23.1 (14.3-34.7)	23.2 (13.6-36.0)	23.2 (14.0-35.9)
No of sit to upright transitions	53.7 (13.8)	53.6 (13.5)	52.3 (14.1)	53.2 (13.8)
Device wear time (min)	941.7 (53.0)	940.4 (56.3)	950.4 (55.2)	944.0 (54.7)
No of valid days	7.39 (1.25)	7.26 (1.33)	7.25 (1.49)	7.30 (1.36)
Daily during work hours:				
Sitting (min)	358.8 (65.3)	356.4 (71.1)	358.1 (67.6)	357.8 (67.9)
Prolonged (≥30 min) sitting (min)	193.7 (86.3)	183.2 (92.5)	194.4 (85.2)	190.5 (88.1)
Median (IQR) standing (min)	69.9 (52.1-98.5)	73.4 (53.6-100.3)	73.5 (50.7-100.1)	71.7 (51.5-99.1)
Stepping (min)	40.2 (14.5)	41.3 (14.4)	40.6 (17.1)	40.7 (15.3)
No of steps	3822.7 (1452.2)	3885.7 (1434.3)	3835.3 (1686.2)	3847.4 (1522.7)
Median (IQR) MVPA stepping time (min)	11.3 (6.97-17.1)	10.8 (6.15-17.1)	11.3 (5.65-17.8)	11.2 (6.29-17.6)
No of sit to upright transitions	27.5 (10.4)	28.2 (11.0)	25.8 (9.48)	27.2 (10.4)
Device wear time (min)	482.5 (45.3)	484.7 (52.4)	482.4 (52.6)	483.2 (50.0)
No of valid days	4.82 (1.32)	4.76 (1.30)	4.81 (1.37)	4.79 (1.33)

CI=confidence interval; IQR=interquartile range; HbA1c=glycated haemoglobin; MVPA=moderate to vigorous physical activity.

The percentage of time participants spent sitting, standing, and stepping was 64.2% (SD 8.3%), 24.3% (SD 7.0%), and 11.5% (SD 3.3%) of daily wear time, and 74.3% (SD 11.7%), 17.5% (SD 10.7%), and 8.5% (SD 3.2%) of work time. More than half of the sitting time was accrued in prolonged bouts (≥30 minutes) (daily: 51.9% (SD 12.1%); work hours: 51.5% (SD 19.0%)).

### Summary of intervention delivery

Training of workplace champions was attended by those representing 51 out of the 52 intervention clusters; by the end of the study, however, 21% of intervention clusters had no workplace champion owing to drop-out. Across both intervention arms, 79.1% of participants completed some or all of the online education, 63.5% of clusters reported sending ≥75% of the monthly emails over the 12 month period, 53.8% of clusters delivered all three challenges (86.5% initiating at least one), 56% of clusters had both group catch-up sessions (82% having at least one), a third of participants reported using self-monitoring and prompt tools, and 82.9% of participants in the SWAL plus desk group at 12 month follow-up reported using their desk at least a few times a week.

### Primary outcome: Change in daily sitting time at 12 month follow-up

**Fig 2:**
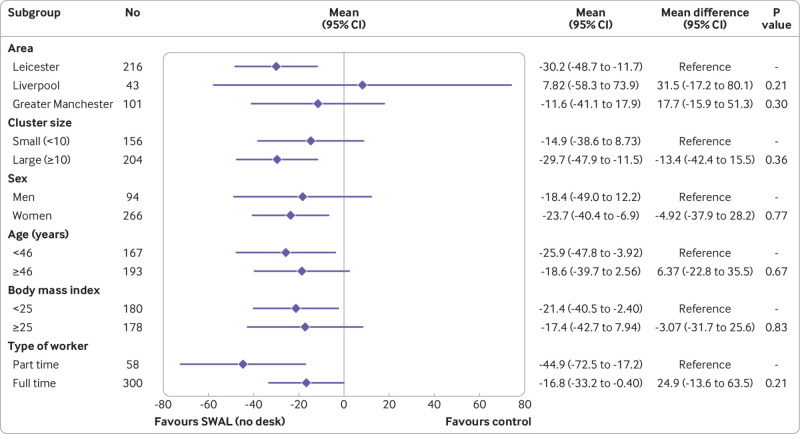
Adjusted difference in average daily sitting time (min/day) at 12 months for SMART Work and Life (SWAL) group

**Fig 3:**
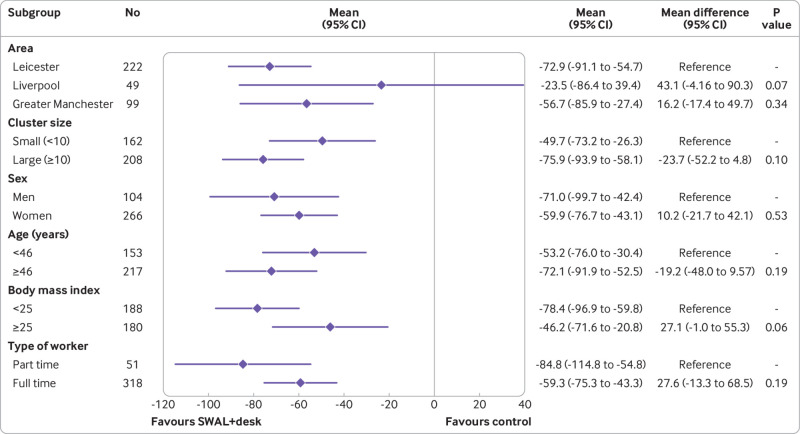
Adjusted difference in average daily sitting time (min/day) at 12 months for SMART Work and Life (SWAL) plus desk group

In the complete case analysis, the SWAL and SWAL plus desk groups sat for 22.2 min/day (95% confidence interval −38.8 to −5.7 min/day, P=0.003) and 63.7 min/day (−80.1 to −47.4 min/day, P<0.001) less than the control group at 12 month follow-up (table 2). Similar results were seen in the sensitivity analyses for intention to treat, per protocol, standardising the waking day, and number of valid activPAL days required (see supplementary table 2). No significant interaction effects were found for either intervention group for any of the subgroups (fig 2 and fig 3). The intervention effects were consistent across age and BMI (see supplementary tables 3 and 4).

**Table 2:** Changes in daily activPAL assessed outcomes (min/day) using data from any valid days at three and 12 months in participants randomised to the SMART Work and Life (SWAL) intervention with or without a desk or to usual practice (control)

Outcomes	Mean (SD) at baseline				Mean (SD) change from baseline to follow-up				Adjusted mean difference at follow-up (95% CI); P value		
	Control	SWAL	SWAL+desk		Control	SWAL	SWAL+desk		SWAL *v* control	SWAL+desk *v* control	SWAL+desk *v* SWAL
**Primary outcome**											
Sitting time 12 month follow-up*†	596.5 (84.1)	601.7 (80.9)	610.4 (78.7)		15.6 (75.0)	−9.4 (80.5)	−53.7 (79.1)		−22.2 (−38.8 to −5.7); 0.003†	−63.7 (−80.1 to −47.4); <0.001†	−41.7 (−56.3 to −27.0), <0.001
**Secondary outcomes**											
Sitting time: 3 month follow-up‡§	599.9 (83.7)	606.3 (81.2)	608.4 (81.1)		−3.5 (75.9)	−27.5 (87.2)	−68.5 (78.1)		−20.0 (−34.9 to −5.0); 0.009	−62.7 (−77.6 to −47.8); <0.001	−43.4 (−60.4 to −26.3), <0.001
Prolonged sitting: 3 month follow-up‡§	314.2 (99.5)	315.9 (92.2)	322.4 (106.0)		8.7 (81.0)	−25.7 (85.4)	−41.9 (83.6)		−32.1 (−47.8 to −16.4); <0.001	−47.4 (−63.0 to −31.8); <0.001	−15.9 (−32.9 to 1.1); 0.07
Prolonged sitting: 12 month follow-up*†	308.7 (101.0)	311.7 (89.3)	324.0 (102.4)		24.9 (74.8)	−5.5 (82.7)	−29.2 (77.8)		−30.5 (−45.3 to −15.7); <0.001	−50.3 (−64.9 to −35.7); <0.001	−19.9 (−35.1 to −4.7); 0.010
Standing time: 3 month follow-up‡§	234.3 (66.2)	225.6 (70.1)	233.6 (71.2)		2.8 (51.3)	9.5 (57.7)	51.1 (64.6)		5.5 (−7.5 to 18.4); 0.41	47.2 (34.3 to 60.2); <0.001	NA
Standing time: 12 month follow-up*†	238.4 (66.2)	229.1 (71.9)	232.1 (68.6)		−5.6 (50.7)	0.1 (60.8)	32.8 (65.6)		6.0 (−6.4 to 18.4); 0.34	39.0 (26.8 to 51.3); <0.001	NA
Stepping time: 3 month follow-up‡§	110.0 (33.0)	109.2 (30.7)	110.3 (33.3)		−3.5 (22.1)	0.5 (26.6)	−1.5 (24.2)		4.7 (−0.5 to 9.8); 0.07	3.0 (−2.1 to 8.1); 0.25	NA
Stepping time: 12 month follow-up*†	112.8 (33.1)	111.7 (30.5)	110.9 (32.9)		−4.7 (20.9)	−1.0 (26.1)	−1.0 (26.9)		4.6 (−0.4 to 9.6); 0.07	4.1 (−0.8 to 9.1); 0.10	NA

SD=standard deviation; CI=confidence interval; NA= Not applicable because the test was not conducted as one intervention group versus control group was not significant.

* Control 26 clusters (183 participants), SWAL 27 (177), SWAL plus desk 25 (187).

† ≥1 valid day at baseline and 12 months. Adjusted for respective average daily outcome at baseline, average wear time of monitor during waking hours across baseline and 12 months, and stratification factors of area (Leicester, Liverpool, Greater Manchester) and cluster size category (small <10, large ≥10).

‡ Control 26 clusters (210 participants), SWAL (27 (200), SWAL plus desk 25 (202).

§ ≥1 valid day at baseline and three months. Adjusted for respective average daily outcome at baseline, average wear time of monitor during waking hours across baseline and three months, and stratification factors of area (Leicester, Liverpool, Greater Manchester) and cluster size category (small <10, large ≥10).

### Secondary outcomes

#### Comparison of interventions

The SWAL plus desk group sat for 41.7 minutes less per day (95% confidence interval −56.3 to −27.0 min/day, P<0.001) than the SWAL group (table 2).

#### Key activPAL assessed secondary outcomes

The SWAL group showed favourable changes compared with the control group in daily sitting time at three months and daily prolonged sitting time at three and 12 months when reported across any valid day or days (table 2), prolonged sitting time during work hours at three and 12 months (table 3), daily sitting time and daily prolonged sitting time on workdays at three and 12 months (table 4), and daily stepping time at three months on workdays (table 4). The SWAL plus desk group showed favourable changes compared with the control group in daily sitting time at three months, daily prolonged sitting time, and daily prolonged standing time at three and 12 months when reported across any valid day or days (table 2), and in sitting time, prolonged sitting time, and standing time at three and 12 months during work hours (table 3) and on workdays (table 4). Additionally, favourable changes in stepping time at 12 months during work hours were observed (table 3 and table 4). No differences were found between groups for any of the outcome variables on non-workdays (table 5).

**Table 3:** Changes in activPAL assessed secondary outcomes (min/work hours) during work hours at three and 12 months in participants randomised to the SMART Work and Life (SWAL) intervention with or without a desk or to usual practice (control)

Outcomes	Mean (SD) at baseline				Mean (SD) change from baseline to follow-up				Adjusted mean difference at follow-up (95% CI); P value		
	Control	SWAL	SWAL+desk		Control	SWAL	SWAL+desk		SWAL *v* control	SWAL+desk *v* control	SWAL+desk *v* SWAL
Sitting time: 3 month follow-up*†	359.7 (63.6)	360.3 (68.85)	355.8 (66.5)		−7.1 (62.5)	−24.8 (62.1)	−82.6 (77.9)		−13.00 (−29.5 to 3.6); 0.13	−74.3 (−90.8 to −57.7); <0.001	NA
Sitting time: 12 month follow-up‡§	356.5 (65.6)	358.0 (71.5)	358.6 (68.1)		2.2 (61.1)	−12.8 (71.0)	−56.4 (85.5)		−13.4 (−29.0 to 2.2); 0.09	−57.9 (−73.3 to −42.5); <0.001	NA
Prolonged sitting: 3 month follow-up*†	191.7 (87.0)	189.8 (93.3)	189.3 (84.7)		−5.3 (68.2)	−28.1 (69.8)	−60.1 (72.6)		−20.5 (−34.6 to −6.4); 0.004	−53.4 (−67.5 to −39.3); <0.001	-33.3 (−48.1 to −18.4); <0.001
Prolonged sitting: 12 month follow-up‡§	186.9 (86.8)	186.9 (90.1)	193.7 (85.4)		8.7 (68.9)	−13.8 (78.00)	−42.0 (69.4)		−21.6 (−35.7 to −7.6); 0.003	−47.7 (−61.6 to −33.8); <0.001	-25.5 (−39.0 to −12.0); <0.001
Standing time: 3 month follow-up*†	85.6 (53.4)	85.6 (55.0)	85.6 (56.0)		6.1 (52.1)	12.3 (49.0)	82.9 (71.4)		4.7 (−10.9 to 20.3); 0.55	74.4 (58.7 to 89.9); <0.001	NA
Standing time: 12 month follow-up‡§	88.4 (55.1)	86.8 (57.0)	83.7 (54.9)		−3.2 (50.3)	11.1 (60.9)	58.5 (76.5)		13.0 (−0.9 to 26.8); 0.07	58.8 (45.1 to 72.5); <0.001	NA
Stepping time: 3 month follow-up*†	40.5 (13.9)	41.2 (14.1)	41.9 (17.4)		0.1 (14.9)	2.0 (15.5)	2.5 (15.8)		2.4 (−1.1 to 5.9); 0.18	2.8 (−0.7 to 6.3); 0.12	NA
Stepping time: 12 month follow-up‡§	41.2 (14.1)	42.0 (14.4)	41.0 (16.7)		−1.5 (14.0)	2.3 (14.9)	4.6 (19.9)		3.4 (−0.2 to 7.1); 0.06	5.4 (1.8 to 9.0); 0.003	NA

SD=standard deviation; CI=confidence interval; NA= Not applicable because the test was not conducted as one intervention group versus control was not significant.

* Control 26 clusters (186 participants), SWAL 26 (175), SWAL+desk 25 (176).

† ≥1 valid workday during work hours at baseline and three months. Adjusted for respective average work hours outcome at baseline, average wear time of monitor during work hours across baseline and three months, and stratification factors of area (Leicester, Liverpool, Greater Manchester) and cluster size category (small <10, large ≥10).

‡ Control 26 clusters (176 participants), SWAL 26 (167), SWAL+desk 25 (177).

§ ≥1 valid workday during working hours at baseline and 12 months. Adjusted for respective average work hours outcome at baseline, average wear time of monitor during work hours across baseline and 12 months, and stratification factors of area (Leicester, Liverpool, Greater Manchester) and cluster size category (small <10, large ≥10).

**Table 4:** Changes in daily activPAL assessed outcomes (min/day) on workdays at three and 12 months (primary outcome) in participants randomised to the SMART Work and Life (SWAL) intervention with or without a desk or to usual practice (control)

Outcomes	Mean (SD) at baseline				Mean (SD) change from baseline to follow-up				Adjusted mean difference at follow-up (95% CI) | P-value		
	Control	SWAL	SWAL+desk		Control	SWAL	SWAL+desk		SWAL *v* Control	SWAL+desk *v* Control	SWAL+desk *v* SWAL
Sitting time: 3 month follow-up*†	645.2 (88.5)	647.9 (81.7)	644.1 (88.5)		−5.5 (83.0)	−31.0 (85.4)	−92.4 (84.2)		−20.4 (−39.5 to −1.3); 0.04	−85.8 (−104.9 to −66.7); <0.001	-65.2 (−86.1 to −44.2); <0.001
Sitting time: 12 month follow-up‡§	642.2 (88.3)	642.4 (83.4)	645.8 (87.7)		7.0 (83.2)	−15.9 (88.0)	−67.5 (95.3)		−19.6 (−36.8 to −2.5); 0.03	−71.1 (−87.9 to −54.3); <0.001	-51.4 (−69.1 to −33.8); <0.001
Prolonged sitting: 3 month follow-up*†	337.4 (113.4)	342.1 (113.0)	340.5 (118.4)		5.2 (94.8)	−34.0 (93.0)	−64.0 (93.2)		−34.4 (−52.9 to −16.0); <0.001	−67.3 (−85.8 to −48.9); <0.001	-33.7 (−53.0 to −14.4); <0.001
Prolonged sitting: 12 month follow-up‡§	331.8 (114.0)	337.1 (106.8)	344.5 (117.5)		19.0 (86.8)	−11.9 (96.7)	−41.6 (85.3)		−26.9 (−44.1 to −9.6); 0.002	−54.6 (−71.5 to −37.7); <0.001	-27.6 (−44.6 to −10.6); <0.001
Standing time: 3 month follow-up*†	219.3 (72.9)	212.1 (73.7)	222.3 (78.9)		3.4 (62.2)	13.8 (59.2)	77.5 (78.4)		7.6 (−8.7 to 23.9); 0.36	72.5 (56.2 to 88.8); <0.001	NA
Standing time: 12 month follow-up‡§	223.0 (72.1)	214.2 (75.9)	218.5 (76.5)		−2.2 (61.5)	5.0 (68.8)	50.0 (81.3)		7.00 (−8.1 to 22.1); 0.36	53.0 (38.1 to 67.8); <0.001	NA
Stepping time: 3 month follow-up*†	106.2 (31.0)	104.8 (30.0)	108.2 (33.6)		−4.0 (20.7)	1.8 (24.3)	−1.00 (24.0)		6.0 (1.5 to 10.4); 0.008	3.6 (−0.9 to 8.0); 0.11	NA
Stepping time: 12 month follow-up‡§	109.0 (31.3)	107.0 (30.5)	107.9 (34.0)		−3.5 (21.3)	0.5 (25.5)	0.1 (28.6)		3.6 (−2.0 to 9.2); 0.20	3.3 (−2.3 to 8.8); 0.25	NA

SD=standard deviation; CI=confidence interval; NA= Not applicable because the test was not conducted as one intervention group versus control was not significant.

Outcomes reported on workdays were not prespecified.

* Control 26 clusters (187 participants), SWAL 26 (175), SWAL+desk 25 (176).

† ≥1 valid workday at baseline and three months. Adjusted for respective average workday outcome at baseline, average wear time of monitor during workday across baseline and three months, and stratification factors of area (Leicester, Liverpool, Greater Manchester) and cluster size category (small <10, large ≥10).

‡ Control 26 clusters (176 participants), SWAL 26 (165), SWAL+desk 25 (177).

§ ≥1 valid workdays at baseline and 12 months. Adjusted for respective average workday outcome at baseline, average wear time of monitor during workdays across baseline and three months, and stratification factors of area (Leicester, Liverpool, Greater Manchester) and cluster size category (small <10, large ≥10).

**Table 5:** Changes in daily activPAL assessed outcomes (min/day) on non-workdays at three and 12 months in participants randomised to the SMART Work and Life (SWAL) intervention with or without a desk or to usual practice (control)

Outcomes	Mean (SD) at baseline				Mean (SD) change from baseline to follow-up				Adjusted mean difference at follow-up (95% CI); P value		
	Control	SWAL	SWAL+desk		Control	SWAL	SWAL+desk		SWAL *v* control	SWAL+desk *v* control	SWAL+desk *v* SWAL
Sitting time: 3-month follow-up*†	518.5 (113.0)	524.5 (107.7)	527.4 (117.3)		−4.9 103.6	−2.7 111.2	−21.2 116.7		6.9 (−13.3 to 27.2); 0.50	−12.3 (−32.8 to −8.1); 0.24	NA
Sitting time: 12-month follow-up‡§	516.0 (110.5)	529.0 (108.2)	528.6 (118.4)		16.6 (114.4)	1.8 (110.6)	−11.4 (106.9)		−4.7 (−26.4 to −17.1); 0.68	−19.8 (−41.3 to −1.8); 0.07	NA
Prolonged sitting: 3-month follow-up*†	269.7 (126.9)	276.4 (112.6)	277.4 (129.6)		6.5 (113.3)	−2.7 (117.9)	−6.5 (120.6)		−3.1 (−25.5 to 19.4); 0.79	−5.8 (−28.4 to 16.8); 0.62	NA
Prolonged sitting: 12-month follow-up‡§	266.5 (123.0)	279.7 (114.2)	283.5 (130.0)		27.3 (126.6)	−4.2 (122.7)	1.2 (122.3)		−21.8 (−48.9 to 5.3); 0.12	15.6 (−42.5 to 11.2); 0.25	NA
Standing time: 3-month follow-up*†	262.7 (87.4)	250.8 (85.8)	266.6 (87.4)		4.6 (66.7)	3.4 (74.6)	8.3 (75.4)		−4.5 (−19.2 to 10.2); 0.55	4.9 (−9.9 to 19.6); 0.52	NA
Standing time: 12-month follow-up‡§	266.7 (85.5)	251.8 (87.2)	261.7 (87.4)		−5.3 (74.5)	−6.6 (79.4)	−2.1 (79.3)		−3.9 (−19.3 to 11.6); 0.63	4.9 (−10.4 to 20.2); 0.53	NA
Stepping time: 3-month follow-up*†	118.4 (46.5)	118.9 (51.3)	113.8 (44.1)		0.3 (39.5)	−2.6 (48.9)	−0.2 (45.7)		−0.6 (−9.9 to 8.7); 0.90	−1.6 (−11.00 to 7.8); 0.74	NA
Stepping time: 12-month follow-up‡§	119.8 (46.0)	120.5 (49.7)	114.7 (44.2)		−2.8 (40.2)	−4.1 (52.6)	−0.6 (43.4)		1.4 (−7.00 to 9.8); 0.74	1.5 (−6.8 to 9.9); 0.72	NA

SD=standard deviation; CI=confidence interval; NA=Not applicable because the test was not conducted as one intervention group versus control was not significant.

Outcomes reported on non-workdays were not prespecified.

* Control 26 clusters (170 participants), SWAL 26 (166), SWAL+desk 25 (162).

† ≥1 valid non-workday at baseline and three months. Adjusted for respective average non-workday outcome at baseline, average wear time of monitor during non-workdays across baseline and three months, and stratification factors of area (Leicester, Liverpool, Greater Manchester) and cluster size category (small <10, large ≥10).

‡ Control 26 clusters (160 participants), SWAL 26 (152), SWAL+desk 25 (156).

§ ≥1 valid non-workday at baseline and 12 months. Adjusted for respective average non-workday outcome at baseline, average wear time of monitor during non-workdays across baseline and three months, and stratification factors of area (Leicester, Liverpool, Greater Manchester) and cluster size category (small <10, large ≥10).

**Fig 4:**
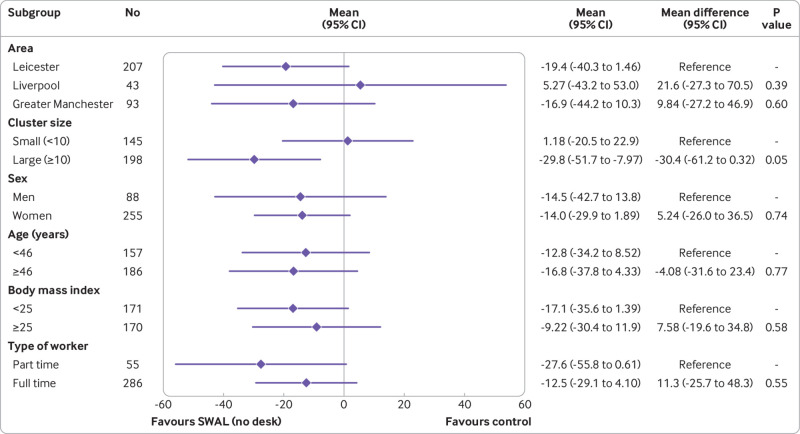
Adjusted difference in average sitting time during work hours (min/day) at 12 months for SMART Work and Life (SWAL) group

**Fig 5:**
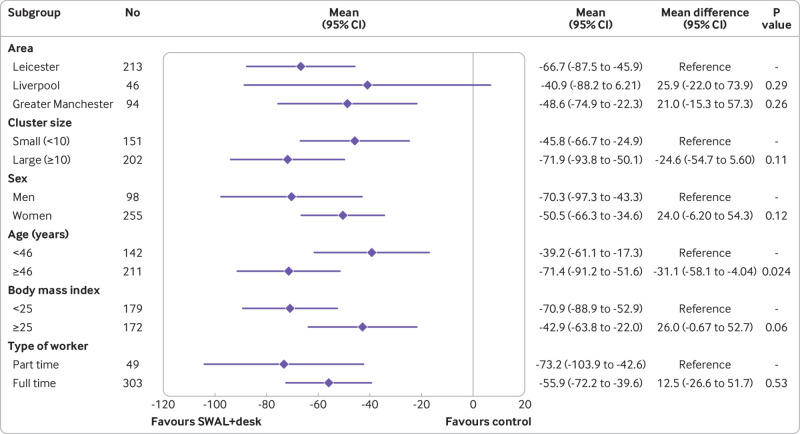
Adjusted difference in average sitting time during work hours (min/day) at 12 months for SMART Work and Life (SWAL) plus desk group

Sensitivity analyses performed on sitting time during work hours showed similar results to the complete case analysis, with the exception of standardising data to an eight hour workday for the SWAL group compared with control group (see supplementary table 5). No significant interaction effects were found for either intervention group for any of the subgroups, with the exception of age for the SWAL plus desk group (fig 4 and fig 5). A significant interaction occurred for age, with the intervention having a greater effect for those aged ≥46 years. When considering age and BMI as continuous variables, a significant interaction effect was found for the SWAL plus desk group for both variables, with average sitting time during work hours decreasing by 1.62 minutes per year increase in age and increasing by 1.20 minutes for each unit increase in BMI (see supplementary tables 3 and 4).

#### Other secondary outcomes

*activPAL*—For daily variables, all groups showed a small reduction in the number of daily steps, time spent in moderate to vigorous physical activity stepping, and the number of sit to upright transitions at three and 12 months (see supplementary table 6). During work hours a similar pattern was seen, apart for number of daily steps, with small favourable changes for both intervention groups compared with the control group at three and 12 months. The pattern of results for each variable was less consistent on workdays and non-workdays.

*Axivity*—For daily and workday variables, no consistent patterns of results or noticeable changes in behaviour were found between groups (see supplementary table 7). During work hours, small favourable changes occurred in light physical activity for the SWAL plus desk group compared with control group at the 12 month follow-up. On non-workdays, small unfavourable changes occurred in light physical activity for the SWAL plus desk group compared with control group at three and 12 months. No changes in sleep duration and efficiency occurred between groups.

*Self-report*—The self-reported sitting and physical activity variables appear to follow a similar pattern to the activPAL assessed sitting and physical variables, with favourable changes in the percentage of time sitting, prolonged sitting, and standing for both intervention groups at the three and 12 month follow-up compared with the control group (table 6). No noticeable changes occurred in other variables between groups.

**Table 6:** Self-reported lifestyle behaviours (secondary outcomes) at baseline and change at three and 12 months compared with baseline in participants randomised to the SMART Work and Life (SWAL) intervention with or without a desk or to usual practice (control)

Lifestyle behaviours	Mean (SD) at baseline				Mean (SD) change at 3 months				Mean (SD) change at 12 months		
	Control	SWAL	SWAL+desk		Control	SWAL	SWAL+desk		Control	SWAL	SWAL+desk
**Self-reported non-work time behaviour**											
Sleep quality*	6.76 (3.25)	6.79 (3.32)	6.95 (3.15)		−0.43 (2.16)	−0.24 (2.42)	−0.46 (2.85)		−0.03 (2.55)	−0.23 (2.74)	−0.17 (2.70)
Sleep duration (h/day)	6.72 (0.97)	6.72 (1.00)	6.63 (0.96)		−0.01 (0.73)	0.06 (0.71)	0.10 (0.70)		−0.06 (0.84)	−0.01 (0.67)	0.02 (0.86)
**Typical working week**											
No of workdays	4.83 (0.54)	4.84 (0.70)	4.81 (0.60)		0.07 (0.54)	−0.02 (0.56)	0.09 (0.61)		0.01 (0.40)	0.04 (0.62)	0.08 (0.63)
No of hours worked per day	7.53 (0.57)	7.62 (0.76)	7.59 (0.74)		−0.05 (0.51)	−0.04 (0.45)	−0.00 (0.59)		−0.03 (0.54)	0.01 (0.69)	−0.05 (0.68)
% of day spent in office	88.1 (15.2)	84.1 (16.6)	88.3 (13.5)		−0.73 (15.7)	−1.48 (16.7)	−1.71 (16.8)		−1.76 (13.6)	−1.64 (16.0)	−4.24 (15.1)
% of day spent at desk	81.3 (15.7)	76.1 (18.7)	81.0 (15.7)		−1.21 (14.7)	−1.09 (15.8)	−1.10 (14.3)		−1.69 (15.2)	−0.98 (16.8)	−2.55 (16.9)
**Self-reported behaviours**											
Workday sitting (%)	81.1 (11.2)	78.5 (13.4)	82.0 (12.2)		−1.99 (11.8)	−5.10 (12.6)	−23.8 (20.5)		−1.34 (11.9)	−5.13 (14.7)	−19.9 (21.2)
Workday standing (%)	6.76 (5.87)	7.64 (6.17)	6.29 (5.70)		1.29 (7.45)	2.79 (7.72)	22.7 (17.9)		0.63 (7.42)	2.92 (10.7)	16.9 (18.9)
Workday walking (%)	12.3 (7.50)	14.0 (9.67)	11.7 (8.19)		0.85 (8.27)	2.30 (9.64)	0.94 (8.78)		0.67 (8.57)	2.33 (9.42)	3.45 (10.5)
Workday prolonged sitting (%)	68.2 (24.7)	67.9 (24.3)	71.8 (23.4)		−1.34 (23.3)	−6.49 (26.6)	−17.5 (28.9)		−2.07 (23.3)	−5.90 (26.7)	−11.5 (28.6)
Sitting while working (min/week)	369.8 (175.8)	358.9 (161.2)	363.7 (170.2)		−35.0 (206.3)	−28.3 (229.8)	−70.8 (207.7)		−9.96(169.7)	−38.1 (226.2)	−62.9 (149.6)
No of sitting breaks per hour	1.64 (1.18)	2.01 (1.50)	1.72 (1.39)		0.37 (1.43)	0.16 (1.63)	0.34 (1.55)		0.31 (1.52)	0.08 (1.84)	0.43 (1.58)
Weekdays (h/weekday):											
Sitting for transport	0.93 (0.67)	1.08 (0.76)	0.93 (0.68)		0.00 (0.87)	−0.04 (0.73)	0.01 (0.65)		−0.04 (0.55)	0.03 (0.85)	0.10 (1.42)
Sitting for TV viewing	1.82 (1.33)	1.70 (1.22)	1.74 (1.17)		−0.13 (1.11)	−0.10 (1.02)	−0.04 (1.00)		−0.25 (1.17)	−0.12 (1.13)	−0.07 (1.11)
Sitting for computer use	1.16 (1.95)	1.03 (1.40)	1.07 (1.61)		−0.13 (1.70)	0.11 (1.46)	0.03 (2.05)		−0.01 (2.29)	0.14 (2.00)	0.22 (2.14)
Sitting other activities	0.65 (0.93)	0.72 (0.87)	0.78 (1.48)		−0.08 (1.03)	−0.38 (1.15)	−0.24 (1.41)		0.07 (1.35)	−0.44 (0.96)	−0.03 (1.67)
All sitting	4.63 (2.42)	4.30 (2.50)	4.60 (3.19)		−0.55 (1.68)	−0.41 (2.63)	−0.67 (3.69)		−0.37 (2.63)	−0.63 (2.48)	0.24 (4.16)
Weekends (h/weekend day):											
Sitting for transport	0.90 (0.75)	1.07 (1.14)	1.10 (1.24)		0.39 (2.34)	−0.06 (1.05)	−0.20 (1.30)		0.15 (0.86)	0.21 (2.07)	−0.22 (1.31)
Sitting for TV viewing	2.79 (1.82)	2.74 (1.90)	2.74 (1.98)		0.13 (1.71)	−0.19 (2.02)	0.12 (2.10)		−0.15 (1.77)	−0.03 (1.51)	0.14 (2.34)
Sitting for computer use	1.38 (1.50)	1.34 (1.30)	1.28 (1.43)		0.18 (1.28)	0.07 (1.72)	0.12 (1.92)		0.09 (1.38)	0.08 (1.48)	0.15 (1.47)
Sitting for other activities	1.26 (1.40)	1.52 (2.01)	1.61 (2.33)		0.11 (2.08)	−0.65 (2.18)	0.03 (3.66)		−0.31 (2.16)	−1.20 (2.18)	−0.20 (2.48)
All sitting	6.40 (2.88)	6.31 (3.46)	6.74 (3.98)		0.32 (3.03)	−0.44 (4.00)	−0.92 (4.15)		−0.22 (3.25)	−0.81 (4.06)	−0.06 (3.95)
Weekly (h/day):											
Sitting for transport	0.92 (0.57)	1.07 (0.68)	0.98 (0.70)		0.11 (0.92)	−0.03 (0.55)	−0.07 (0.62)		0.02 (0.50)	0.06 (0.77)	0.02 (1.16)
Sitting for TV viewing	2.08 (1.31)	1.97 (1.27)	2.04 (1.22)		−0.04 (1.14)	−0.12 (1.08)	−0.01 (1.09)		−0.20 (1.21)	−0.07 (1.04)	−0.03 (1.19)
Sitting for computer use	1.26 (1.63)	1.09 (1.16)	1.11 (1.32)		−0.03 (1.34)	0.12 (1.31)	0.10 (1.66)		−0.03 (1.83)	0.16 (1.59)	0.22 (1.73)
Sitting for other activities	0.79 (0.96)	0.93 (1.11)	1.01 (1.67)		−0.02 (1.25)	−0.44 (1.34)	−0.16 (1.96)		−0.03 (1.53)	−0.69 (1.24)	−0.13 (1.75)
All sitting	5.09 (2.13)	4.74 (2.30)	5.18 (3.12)		−0.35 (1.85)	−0.38 (2.35)	−0.73 (3.36)		−0.39 (2.56)	−0.69 (2.25)	0.35 (3.62)
**Self-reported dietary behaviours**											
Snack frequency (% reporting ≥1/day)	28.3 (70)	33.3 (77)	31.5 (70)		0.6	0.0	−2.7		0.0	2.6	−4.5
Soft drink consumption (% reporting ≥1/day)	11.3 (28)	18.3 (42)	18.7 (42)		0.6	−3.8	−6.4		1.8	2.6	−5.0
Fruit consumption (% reporting ≥1/day)	70.8 (177)	69.6 (160)	68.2 (152)		−2.9	4.8	4.4		1.2	−3.9	5.1
Vegetable consumption (% reporting ≥1/day)	80.8 (202)	72.9 (167)	75.3 (168)		−2.9	9.1	−0.5		−1.8	5.8	2.3
Alcohol intake (Total units/week)	8.70 (8.09)	9.91 (8.58)	9.84 (8.98)		0.31 (5.27)	0.84 (10.2)	−0.78 (7.43)		−0.43 (4.89)	−0.68 (5.75)	−1.63 (7.07)

* Score 0-21 (higher score indicates worse sleep quality).

*Physical health*—No noticeable between group differences were found in the mean changes for any cardiometabolic health variable or fatigue at follow-up (table 7). For musculoskeletal conditions, there appeared to be small favourable changes in the prevalence of, and pain experienced in, the lower extremity in the SWAL plus desk group compared with the control group at 12 months.

**Table 7:** Physical health (secondary outcomes) at baseline, and change at three and 12 months compared with baseline in participants randomised to the SMART Work and Life (SWAL) intervention with or without a desk or to usual practice (control)

	Mean (SD) at baseline				Mean (SD) change at 3 months				Mean (SD change at 12 months		
	Control	SWAL	SWAL+desk		Control	SWAL	SWAL+desk		Control	SWAL	SWAL+desk
**Adiposity**											
Weight (kg)	71.6 (17.1)	75.1 (18.1)	73.8 (17.6)		−0.01 (2.16)	−0.17 (2.87)	−0.03 (2.91)		0.05 (3.25)	0.24 (4.15)	0.31 (4.30)
Waist circumference (cm)	86.6 (13.7)	89.0 (15.0)	89.2 (14.4)		−0.64 (4.85)	−0.03 (5.44)	−1.25 (5.47)		−0.94 (5.92)	−0.61 (6.67)	−1.58 (6.80)
Body fat (%)	32.4 (9.36)	33.7 (9.44)	32.3 (9.27)		0.49 (3.74)	0.01 (3.58)	0.46 (3.33)		−0.07 (4.27)	0.46 (2.91)	0.53 (3.37)
Body mass index	25.8 (5.60)	27.3 (6.42)	26.4 (5.68)		−0.01 (0.78)	−0.07 (1.08)	−0.02 (1.09)		0.01 (1.20)	0.09 (1.53)	0.10 (1.55)
**Blood pressure (mm Hg)**											
Systolic	116.9 (14.5)	119.0 (17.3)	119.2 (16.6)		−1.96 (9.99)	−1.04 (9.93)	−2.09 (11.3)		−1.44 (10.8)	−1.78 (10.4)	−2.08 (11.8)
Diastolic	78.1 (9.46)	79.4 (10.7)	79.9 (11.1)		−1.16 (6.75)	−0.28 (6.29)	−1.96 (7.42)		0.18 (6.91)	0.08 (7.12)	−1.07 (7.85)
Biochemical											
HbA1c (mmol/mol)	33.5 (5.77)	33.8 (4.99)	34.5 (5.41)		1.11 (4.06)	0.40 (2.94)	0.86 (3.38)		2.42 (3.73)	1.67 (3.10)	1.84 (4.10)
HbA1c (%)	5.22 (0.53)	5.24 (0.46)	5.31 (0.50)		0.10 (0.37)	0.04 (0.27)	0.08 (0.31)		0.22 (0.34)	0.15 (0.28)	0.17 (0.37)
Cholesterol (mmol/L):											
Total (mmol/L)	4.64 (1.04)	4.71 (1.07)	4.67 (1.06)		−0.19 (0.80)	−0.05 (0.79)	−0.09 (0.79)		−0.28 (0.88)	−0.29 (0.89)	−0.34 (0.81)
High density lipoprotein (mmol/L)	1.46 (0.38)	1.41 (0.42)	1.42 (0.39)		−0.01 (0.26)	0.01 (0.29)	0.02 (0.20)		−0.05 (0.29)	−0.02 (0.29)	−0.03 (0.23)
Low density lipoprotein (mmol/L)	2.52 (0.96)	2.65 (1.26)	2.56 (1.00)		−0.19 (0.75)	−0.19 (1.15)	−0.21 (0.93)		−0.15 (0.93)	−0.29 (1.28)	−0.29 (0.89)
Triglycerides (mmol/L)	1.19 (0.62)	1.22 (0.59)	1.24 (0.64)		0.01 (0.80)	0.07 (0.61)	0.03 (0.64)		−0.06 (0.72)	0.01 (0.60)	−0.04 (0.58)
Fasting glucose (mmol/L)	5.44 (1.07)	5.44 (0.76)	5.58 (1.04)		0.14 (0.82)	0.02 (0.71)	−0.01 (0.76)		−0.18 (0.83)	−0.15 (0.83)	−0.24 (1.12)
Cardiometabolic risk score*	−0.08 (0.61)	0.01 (0.67)	0.07 (0.67)		0.02 (0.33)	0.02 (0.35)	−0.05 (0.33)		0.01 (0.36)	0.02 (0.41)	−0.04 (0.36)
Fatigue†:											
Physical	8.78 (3.50)	8.83 (3.80)	8.76 (3.35)		−0.22 (3.42)	−0.42 (3.67)	−0.77 (3.66)		−0.12 (3.63)	0.06 (4.35)	−0.25 (3.64)
Mental	5.02 (2.12)	4.99 (2.20)	4.83 (2.03)		−1.27 (2.25)	−0.83 (2.04)	−0.82 (1.92)		−1.14 (2.31)	−1.15 (2.32)	−0.84 (2.15)
Global	13.8 (5.08)	13.8 (5.50)	13.6 (4.98)		−4.27 (7.36)	−2.90 (6.90)	−3.04 (6.44)		−4.06 (7.62)	−3.98 (8.08)	−2.90 (7.02)
**Musculoskeletal conditions**											
Area affected in past 3 months:											
Neck	55.6 (134)	54.8 (126)	54.1 (119)		−9.8	−12.8	−15.9		−6.6	−9.2	−15.8
Lower back	61.6 (151)	58.1 (133)	52.5 (115)		−10.9	−13.4	−8.9		−11.5	–10.7	−8.2
Upper extremity‡	72.9 (180)	72.3 (167)	69.1 (154)		−14.3	−9.1	−10.9		−8.2	−16.7	−11.8
Lower extremity§	65.3 (160)	70.4 (162)	68.2 (150)		−16.9	−18.9	−12.6		−4.1	−12.8	−11.3
Any part	88.8 (221)	92.2 (214)	89.4 (202)		−9.4	−9.5	−6.9		−5.2	−11.4	−7.2
Pain in past 3 months¶:											
Neck	1.82 (2.26)	1.63 (2.09)	1.58 (1.93)		−0.44 (1.86)	−0.25 (1.92)	−0.53 (1.95)		−0.29 (1.83)	−0.13 (1.97)	−0.39 (1.90)
Lower back	2.42 (2.57)	2.32 (2.60)	2.10 (2.50)		−0.52 (2.50)	−0.77 (2.47)	−0.55 (2.13)		−0.44 (2.44)	−0.50 (2.44)	−0.43 (2.69)
Upper extremity	2.33 (2.11)	2.26 (2.04)	2.27 (2.15)		−0.39 (1.97)	−0.14 (2.36)	−0.43 (2.20)		−0.24 (1.82)	−0.50 (2.38)	−0.37 (2.21)
Lower extremity	2.18 (2.37)	2.50 (2.42)	2.45 (2.23)		−0.30 (2.12)	−0.45 (2.59)	−0.50 (2.39)		0.07 (2.35)	−0.13 (2.56)	−0.47 (2.53)
Any part	2.95 (1.93)	3.14 (1.87)	3.08 (1.86)		−0.26 (1.81)	−0.24 (2.32)	−0.27 (2.21)		−0.12 (1.77)	−0.29 (2.32)	−0.24 (2.42)

* Outcome not prespecified.

† Mental fatigue scale 0-12 (higher score indicates greater fatigue), physical fatigue scale 0-21 (higher score indicates higher fatigue), global fatigue scale 0-33 (higher score indicates higher fatigue).

‡ Shoulder, upper back, elbow, or wrist/hand.

§ Hip/thigh, knew, or ankle/foot.

¶ 0=no pain; 9=most pain can imagine (higher score indicates greater pain).

*Psychological health*—There appeared to be small favourable changes in stress and wellbeing in both intervention groups compared with the control group at three and 12 months (table 8). For other outcomes, no noticeable between group differences were found.

**Table 8:** Psychological health (secondary outcomes) at baseline and change at three and 12 months compared with baseline in participants randomised to the SMART Work and Life (SWAL) intervention with or without a desk or to usual practice (control)

	Mean (SD) at baseline				Mean (SD) change at 3 months				Mean (SD) change at 12 months		
	Control	SWAL	SWAL+desk		Control	SWAL	SWAL+desk		Control	SWAL	SWAL+desk
Anxiety*	7.44 (4.05)	7.36 (4.14)	6.99 (3.90)		0.11 (2.69)	−0.47 (3.17)	−0.38 (2.78)		−0.16 (2.91)	−0.24 (3.10)	−0.45 (3.13)
Depression†	4.28 (3.44)	3.98 (3.43)	3.94 (3.17)		−0.09 (2.16)	−0.04 (2.72)	−0.20 (2.90)		−0.15 (2.68)	−0.20 (3.04)	−0.31 (2.73)
Stress†	15.9 (6.52)	16.4 (7.02)	16.1 (6.67)		0.43 (5.10)	−0.26 (5.41)	−0.58 (5.64)		0.44 (5.16)	−0.12 (5.55)	−0.24 (5.57)
Wellbeing‡	54.7 (20.1)	54.0 (20.3)	55.4 (19.6)		0.05 (13.9)	2.46 (16.3)	2.37 (17.0)		0.69 (14.5)	2.06 (19.7)	2.12 (15.8)
Positive affect§	30.3 (8.41)	29.7 (8.46)	30.3 (7.88)		−0.30 (6.61)	0.66 (6.59)	0.05 (6.93)		−0.26 (6.91)	0.46 (7.38)	−0.21 (6.76)
Negative affect¶	16.8 (6.87)	16.6 (7.04)	16.1 (6.19)		−0.45 (5.32)	0.34 (6.24)	0.03 (5.89)		0.17 (6.56)	0.24 (6.97)	−0.45 (5.99)
Quality of life:											
Health utility score**	0.90 (0.10)	0.88 (0.13)	0.89 (0.10)		−0.01 (0.09)	0.00 (0.09)	0.01 (0.09)		−0.01 (0.09)	0.00 (0.12)	0.00 (0.10)
Health state score††	74.8 (15.8)	72.9 (16.3)	74.6 (16.0)		1.02 (13.8)	0.87 (12.4)	1.69 (14.6)		2.30 (15.7)	2.24 (14.3)	1.79 (13.9)

* 0=most positive response, 3=most negative response, score 0-21 (higher score indicates greater symptoms).

† 0=never, 4=very often, score 0-40 (higher score indicates greater stress).

‡ Wellbeing no time=1, all of the time=5, score 0-100 (higher score indicates higher wellbeing).

§ Score 10-50 (higher score indicates higher positive affect).

¶ Score 10-50 (lower score indicates lower negative affect).

** EQ-5D-5L time trade-off value set: −0.281 to 1.000 (higher score indicates higher health utility).

†† 0 represents the worst perceived health and 100 represents the best perceived health (higher score indicates better perceived health).

*Work related outcomes*—Small favourable changes in vigour were found for both intervention groups compared with the control group at 12 months and in organisational social norms and all types of support in the SWAL plus desk group at the three and 12 month follow-up compared with the control group (table 9). No noticeable between group differences were found in the mean changes in job performance and satisfaction, occupational fatigue recovery, workload and relations, social community, and absenteeism episodes.

**Table 9:** Work related outcomes (secondary outcomes) at baseline and change at three and 12 months compared with baseline in participants randomised to the SMART Work and Life (SWAL) intervention with or without a desk or to usual practice (control)

	Mean (SD) at baseline				Mean (SD) change at 3 months				Mean (SD) change at 12 months		
	Control	SWAL	SWAL+desk		Control	SWAL	SWAL+desk		Control	SWAL	SWAL+desk
Work engagement*:											
Vigour	3.31 (1.33)	3.42 (1.27)	3.30 (1.25)		0.09 (0.86)	0.10 (0.95)	0.16 (0.87)		0.05 (0.96)	0.17 (0.93)	0.16 (0.98)
Dedication	4.13 (1.22)	4.23 (1.23)	4.09 (1.16)		−0.09 (0.75)	−0.10 (0.77)	0.02 (0.75)		−0.11 (0.85)	−0.05 (0.84)	−0.02 (0.94)
Absorption	4.14 (1.13)	4.22 (1.11)	4.16 (1.06)		0.02 (0.90)	−0.07 (0.82)	0.05 (0.80)		0.06 (0.87)	−0.08 (0.93)	0.08 (0.94)
Overall	3.86 (1.10)	3.96 (1.08)	3.85 (1.01)		0.00 (0.66)	−0.03 (0.67)	0.08 (0.61)		−0.00 (0.73)	0.01 (0.71)	0.07 (0.78)
Job performance†	5.52 (1.01)	5.54 (1.05)	5.53 (0.93)		0.06 (0.82)	−0.02 (0.96)	−0.11 (0.99)		−0.12 (0.94)	−0.10 (1.03)	−0.06 (1.10)
Job satisfaction‡	4.80 (1.38)	4.97 (1.29)	4.89 (1.24)		−0.02 (0.98)	0.04 (0.94)	−0.10 (1.04)		−0.07 (1.17)	0.01 (1.17)	−0.14 (1.27)
Occupational fatigue recovery§	0.45 (0.28)	0.46 (0.29)	0.44 (0.28)		0.01 (0.21)	0.00 (0.22)	−0.00 (0.23)		0.01 (0.23)	0.00 (0.25)	−0.03 (0.25)
Workload and relations¶:											
Demands	2.70 (0.75)	2.70 (0.66)	2.81 (0.74)		0.04 (0.52)	0.01 (0.52)	−0.06 (0.51)		0.04 (0.63)	0.09 (0.58)	−0.04 (0.52)
Control	3.82 (0.71)	3.87 (0.67)	3.81 (0.63)		−0.05 (0.59)	−0.04 (0.53)	−0.00 (0.50)		0.03 (0.53)	−0.06 (0.56)	0.03 (0.57)
Support	3.83 (0.69)	3.87 (0.80)	3.68 (0.83)		−0.11 (0.51)	−0.08 (0.62)	−0.01 (0.57)		−0.05 (0.68)	−0.06 (0.65)	−0.08 (0.70)
Organisational social norms**	3.84 (0.57)	3.93 (0.54)	3.84 (0.54)		−0.01 (0.41)	−0.02 (0.47)	0.25 (0.45)		−0.03 (0.54)	0.00 (0.55)	0.20 (0.63)
Social community††	1.81 (0.73)	1.87 (0.75)	1.96 (0.73)		0.14 (0.64)	−0.05 (0.61)	−0.05 (0.58)		0.13 (0.70)	−0.04 (0.73)	0.07 (0.74)
Support‡‡::											
Organisation	2.91 (1.16)	3.05 (1.17)	2.95 (1.13)		−0.05 (1.31)	−0.01 (1.27)	0.61 (1.17)		−0.04 (1.28)	0.01 (1.32)	0.55 (1.33)
Manager	3.11 (1.24)	3.24 (1.23)	3.09 (1.16)		−0.12 (1.21)	−0.10 (1.34)	0.55 (1.19)		−0.12 (1.28)	0.10 (1.34)	0.32 (1.51)
Colleagues	3.25 (1.15)	3.27 (1.18)	3.24 (1.20)		−0.18 (1.30)	0.08 (1.32)	0.63 (1.29)		−0.08 (1.31)	0.20 (1.31)	0.48 (1.37)
Family	3.40 (1.23)	3.44 (1.21)	3.39 (1.18)		0.00 (1.37)	−0.18 (1.38)	0.18 (1.24)		−0.16 (1.37)	0.08 (1.47)	0.15 (1.35)
Work limitations§§:											
Time management	1.71 (0.84)	1.75 (0.82)	1.63 (0.79)		−0.06 (0.76)	0.02 (0.80)	0.03 (0.78)		0.02 (0.79)	0.09 (0.92)	0.07 (0.81)
Physical demands	1.68 (0.87)	1.72 (0.93)	1.63 (0.85)		0.01 (1.07)	0.03 (1.08)	−0.03 (0.83)		0.03 (1.00)	0.18 (1.06)	0.01 (1.03)
Mental-interpersonal demands	1.55 (0.65)	1.65 (0.73)	1.61 (0.74)		0.06 (0.58)	−0.01 (0.78)	−0.02 (0.83)		−0.01 (0.68)	0.04 (0.83)	−0.04 (0.84)
Output demands	1.59 (0.78)	1.69 (0.88)	1.70 (0.94)		0.09 (0.81)	−0.06 (0.90)	−0.08 (0.96)		0.06 (0.88)	0.04 (0.99)	−0.07 (0.94)
Overall	1.58 (0.54)	1.63 (0.62)	1.60 (0.62)		0.04 (0.53)	−0.00 (0.62)	−0.02 (0.58)		0.01 (0.53)	0.10 (0.68)	−0.00 (0.61)
Absenteeism (self-report)	1.57 (4.58)	0.95 (3.71)	0.82 (2.38)		NA	NA	NA		−0.57 (4.90)	0.37 (6.73)	0.86 (7.77)
Absenteeism (records)											
Episodes	0.80 (1.14)	0.89 (1.47)	0.75 (0.96)		NA	NA	NA		−0.01 (1.37)	−0.06 (1.20)	−0.01 (1.14)
Duration	4.93 (14.2)	4.22 (9.88)	3.86 (7.89)		NA	NA	NA		−0.34 (20.8)	0.87 (14.0)	2.16 (14.4)

NA=Not applicable as not measured at that time point.

* 0=never, 6=always (higher score indicates greater work engagement).

† 1=dissatisfied, 7=extremely satisfied.

‡ 1=very poorly, 7=extremely well.

§ 1=yes=1, 0=no.

¶ 1=never, 5=always demands (higher score indicates greater demands); control, higher score indicates greater control; support, higher score indicates greater support.

** 1=strongly disagree, 5=strongly agree (higher score indicates better social norms).

†† 1=always, 5=never or hardly ever (higher score indicates less social cohesion).

‡‡ 1=not supportive, 5=extremely supportive (higher score indicates greater support).

§§ 1=most positive response, 5=most negative response; time management, higher score indicates worse time management; physical demands, higher score indicates greater physical demands; mental-interpersonal demands, higher score indicates greater mental-interpersonal difficulty; output demands, higher score indicates greater output demands; overall, higher score indicates worse overall productivity.

### Adverse events

Overall, 22.4% (n=169) of participants reported at least one adverse event during the study. Of these, 24.9% (n=55) were related to irritation from wearing the activPAL or Axivity devices, 0.9% (n=2) to the intervention (back pain), 2.3% (n=5) to the measurement session (pain or feeling unwell during blood test), and 71.9% (n=159) were unrelated to the study.

## Discussion

This study aimed to evaluate the effectiveness of the SMART Work and Life (SWAL) intervention, delivered with and without a height adjustable desk, and by workplace champions, compared with a control group. Both intervention groups (with and without a height adjustable desk) were shown to be effective, with the SWAL group sitting for 22 minutes less daily and the SWAL plus desk group for 64 minutes less daily than the control group at 12 months. The SWAL plus desk group sat for 42 minutes less daily than the SWAL group, showing it to be more effective. Time spent in prolonged sitting was lower in both intervention groups compared with the control group. Reductions in sitting time were largely replaced by increases in standing time, and these changes occurred on workdays and during work hours. Furthermore, the magnitude of behaviour change was similar across the three and 12 month follow-up, indicating that behaviour change was maintained during the course of the study. Finally, the SWAL plus desk intervention appeared more effective during work hours for those older than the median age (≥46 years).

Results were suggestive of small improvements in stress and wellbeing at three and 12 months and vigour at 12 months for both intervention groups, and in organisational social norms, all types of support, and pain in the lower extremity in the SWAL plus desk group at three and 12 month follow-up compared with the control group. For other health, work, and wellbeing outcomes, no noticeable between group differences were found.

### Comparison with previous research

The results for change in sitting time at the 12 month follow-up for the SWAL plus desk group are consistent with two other large randomised controlled trials evaluating multicomponent interventions to promote reductions in sitting that included height adjustable desks.^26^ ^27^ However, the SWAL intervention was solely facilitated by workplace champions, unlike the previous interventions, which were delivered by researchers. One other large, randomised trial without a control group recruited employees from academic, industry, healthcare, and government and compared two intervention groups.^72^ The trial found that the intervention (Stand and Move at Work) when provided with a height adjustable desk was more effective at reducing daily sitting time than the intervention provided on its own by 48 minutes per 16 hour waking day, a similar effect size to the present study (42 min/day). Collectively, however, all previous interventions and SWAL showed no or minimal changes in movement time for the intervention groups, with reductions in sitting being replaced by increased accumulated standing time.

Previous interventions targeting a reduction in sitting at work,^26^ ^27^ ^72^ observed no reductions in sitting time outside of work. To maximise health effects, our intervention encouraged participants to reduce and break up sitting time during and outside of work. Despite this, our results indicate that changes in daily sitting time (ie, work and outside of work) were driven only by changes in sitting time during work hours, suggesting that more research is needed to understand how people can be supported to change behaviour outside of work. A recent review found that it might be possible to reduce leisure time sitting in adults in the medium term by about 30 minutes a day, although this was based on a small number of substantially heterogeneous studies.^74^

The SWAL plus desk intervention resulted in more than a 60 minute difference in daily sitting time compared with the control, from a baseline value of about 10 hours a day. Observational evidence has suggested that these changes are likely to be clinically relevant, with the potential to improve health outcomes. A 2018 meta-analysis suggested an association between sitting time and all cause and cardiovascular mortality, with the strength of association increasing beyond eight hours and six hours of sitting daily, respectively.^75^ Beyond these thresholds, every additional hour spent sitting was associated with a 4-6% higher relative risk of mortality outcomes.^75^ A later harmonised meta-analysis of accelerometer measured sedentary behaviour suggested these estimates, which were largely driven by self-reported data, were likely to have been an underestimate.^76^ From a reference of 7.5 hours daily of sedentary behaviour, the risk of mortality increased steeply beyond 9.5 hours, reaching a hazard ratio of 1.5 at 10 hours and a hazard ratio of 2.1 by 11 hours.^76^ However, although observational associations are well established, the effect of interventions against sedentary behaviour on proximal markers of health in the general population is less certain.^77^ In the current study, we observed no changes in the prespecified cardiometabolic health markers; however, average values were in the healthy ranges for all markers except BMI. A similar recent study performed a subgroup analysis on those participants who had increased fasting glucose levels at baseline and found effects sizes to be larger and clinically meaningful for many cardiometabolic health markers.^72^ This type of subgroup was not in our analysis plan; however, a third of our sample, evenly spread across the three arms, had fasting glucose levels of ≥5.6 mmol/L so could warrant further investigation. The results suggest that the SWAL intervention might have small benefits for individuals and employers in terms of lower stress levels, enhanced feelings of wellbeing and vigour, as well as reduced pain for lower extremity musculoskeletal conditions.

Perceived stress was scored on a scale of 0-40, with higher scores indicating higher stress. On average, the participants in all arms scored in the lower end of the moderate stress category (14-26 points),^78^ with averages of 15.9, 16.4, and 16.1 for the control, SWAL, and SWAL plus desk groups, respectively. Although small positive changes were observed for stress in both intervention groups compared with the control group at the three month and 12 month follow-ups, the effect sizes were small, with a 0.7 and 1.0 point difference between control and SWAL and control and SWAL plus desk, respectively, at three months, and a 0.6 and 0.7 point difference at 12 months, respectively. All groups would therefore still be in the moderate stress category. Although it is recommended to include a measure of stress in workplace interventions for reducing sitting time at work,^24^ this has not been done by large scale randomised controlled trials,^26^ ^27^ ^72^ and therefore research results are limited to compare with our findings. Our results are consistent with one study that did include a measure of stress and evaluated the effect of organisational level strategies to reduce sitting time in a group of desk based office workers and found statistically significant but small changes in stress at 12 months.^79^

Similarly, the observed differences in wellbeing were small and not considered clinically meaningful. A clinically relevant change on the World Health Organization-five Well-Being Index (WHO-5) is considered to be 10 points.^55^ We observed a difference of about 2.5 and 2.0 for both intervention groups compared with the control group at the three and 12 month follow-ups, with wellbeing increasing in the intervention groups but remaining constant over time in the control group. Wellbeing is scored on a scale of 0-100, with 0 being the worst imaginable wellbeing to 100 representing the best, with all three randomisation groups scoring between 54.0 and 55.4 on the scale at baseline. A recent systematic review found that conclusive evidence for physical activity interventions improving wellbeing in working age adults is lacking^80^; however, more meaningful changes may be seen in those who had poorer wellbeing at baseline (<52% measured using the WHO-5).^81^ Furthermore, a physical activity behavioural intervention in those with a higher BMI showed a significant increase in WHO-5 score (change of 7.4), but this did not exceed 10, the clinically relevant difference.^82^

Previous research has suggested that interventions to reduce sitting have the potential to be beneficial for work engagement.^83^ Work engagement is defined as “a positive, fulfilling, work-related state of mind that is characterised by vigour, dedication, and absorption.”^84^ We found small positive changes to vigour in both intervention groups compared with the control group at 12 months. The effect sizes were, however, smaller than for our previous intervention,^26^ which showed statistically significant differences in vigour between the control and intervention at the six and 12 month follow-ups. Other smaller, short term sitting reduction interventions have either found small, non-significant differences in favour of the intervention, or no changes.^85^ It has been argued, however, that as long as the intervention is not leading to a negative impact on work related outcomes such as work engagement and productivity as a result of standing and moving more throughout the workday, then this could be interpreted as a positive finding.^85^

A systematic review of workplace interventions for reducing sitting concluded that effects on musculoskeletal symptoms are unclear, with studies showing either small improvements, worse symptoms, or no change.^24^ In our study, we found that in all areas reported, prevalence of musculoskeletal problems and pain ratings decreased in all groups at both three month and 12 month follow-ups, with no clear trend for one group showing greater improvements, with the exception of prevalence of, and pain in, the lower extremity for the SWAL plus desk group. The small effect sizes we observed were similar to a previous sitting reduction intervention using the Nordic Questionnaire pain scale.^86^ It is clear from our results, however, that musculoskeletal symptoms and pain did not increase, particularly in the SWAL plus desk group despite the increase in standing time. Previous research has suggested that substantial occupational standing is associated with prevalence of low back and lower extremity symptoms.^87^

We are conducting a health economics analysis that will highlight whether the behaviour change observed for each group and the impact on secondary outcomes deems the intervention, with and without a height adjustable desk, to be cost effective. Further research is also needed to understand relative return on investment.

A key element of the intervention was the transfer of intervention delivery from the research team to a workplace champion, an approach that allows for potential scale-up of the intervention.^88^ The process evaluation of SWAL will provide insight into organisation, workplace champion, and participant experiences of the intervention, intervention fidelity, and any perceived benefits. The brief data presented in this paper on implementation of the intervention highlight that workplace champions and participants engaged with our intervention, but this varied considerably across clusters and by intervention strategy. These findings can be used to inform further programme adaptations to ensure suitability for sustainable delivery by a range of workplaces.

### Strengths and limitations of this study

Our study has several strengths and limitations. Firstly, the study was a large cluster randomised controlled trial evaluating interventions to reduce sitting time. The intervention was delivered by workplace champions within local councils, mimicking a real world intervention delivery and therefore improving scalability. To test the concept of having the desk rather than testing a specific desk, we gave the intervention group that received the height adjustable desk a choice of model, size, and colour. Although we originally planned to also conduct a 24 month follow-up assessment and were unable to do so because of the covid-19 pandemic, only a handful of studies have evaluated these types of intervention beyond three months; therefore, our 12 month data still provide useful evidence on the medium term impact of our intervention. Our primary outcome and key secondary outcomes were assessed with accelerometry, which reduces bias associated with self-report. However, although wearers are blinded to the accelerometer data, reactivity could have occurred, as participants were aware of the purpose of the accelerometer. Despite using a validated set of questionnaires to measure psychological health and important work related outcomes, reporting bias was possible. For the primary analysis at the 12 month follow-up, data were available for 72% of the randomised participants. Our sample size was sufficiently large enough to account for this proportion. Moreover, when we compared the characteristics of those who had data for the primary analysis with those who did not, we found no evidence of differences. We performed several sensitivity analyses (intention to treat, per protocol, and the effect of differing activPAL days and standardising data to a 16 hour waking day), which confirmed the findings of our primary analysis, indicating the robustness of our results. Finally, we recruited participants across six councils from three areas of England, which increases generalisability compared with previous studies; the participants worked in local government, however, so results may not be generalisable to other employment sectors. It will be important to evaluate further iterations of the intervention across a range of industries.

### Conclusion and future research

The SWAL intervention, delivered with and without a height adjustable desk and by workplace champions, was effective. Both intervention groups (SWAL with and without a desk) sat less than the control group (usual practice) in the short and medium term; furthermore, those receiving the height adjustable desk alongside the intervention sat less than those receiving the intervention only. Small but non-clinically meaningful improvements were found in stress, wellbeing, vigour, and pain in the lower extremity, with no negative effects on any work related outcomes or musculoskeletal problems. This study was a key research-to-translation step. Areas for future research include exploring how people can best be supported to make changes outside of work as well as to increase time spent moving, conducting implementation studies across different employment sectors, and following participants and organisations for a longer period to investigate behaviour and culture change beyond 12 months, and any associated impacts on health, work, and wellbeing outcomes.

**What is already known on this topic**

Office based workers spend most of their working day sitting and also show high levels of sitting time outside of work
High levels of sitting time are associated with several health related outcomes and premature mortality, with high levels of workplace sitting associated with low vigour and job performance and high levels of presenteeism
Large, long term randomised controlled trials should evaluate interventions for reducing sitting time in the workplace to tackle the gaps in low quality studies

**What this study adds**

The SMART Work and Life (SWAL) intervention (with and without a height adjustable desk) was effective in reducing daily sitting time
The SWAL plus desk intervention was three times was more effective at reducing sitting time than the SWAL intervention
Small, but non-clinically meaningful improvements in stress, wellbeing, and vigour were observed for both intervention groups, as well as pain in the lower extremity in the SWAL plus desk group

## Data Availability

Requests for access to data from the study should be sent to the corresponding author (ce95@le.ac.uk). The study protocol has been published. All proposals requesting data access will need to specify how the data will be used, and all proposals will need approval of the trial co-investigator team before data release.

## Web extra

Extra material supplied by authors

## Notes

Contributors: CLE, SJHB, MJD, DWD, GNH, LJG, TY, FM, SAC, GR, HE, MHG, and ACC obtained funding. All authors contributed to the design of the study. NBJ performed the statistical analysis. BM and SL were involved in data collection and study coordination, supervised by CLE and ACC. CLE produced the first draft of this manuscript and all authors reviewed, edited, and approved the final version. CLE is the guarantor. The corresponding author attests that all listed authors meet authorship criteria and that no others meeting the criteria have been omitted.

The guarantor (CLE) affirms that the manuscript is an honest, accurate, and transparent account of the study bring reported; that no important aspects of the study have been omitted; and that any discrepancies from the study as planned have been explained.

Dissemination to participants and related patient and public communities: The results of the study will be disseminated through an executive summary to the council senior leadership teams and through a newsletter to all participants. A more detailed presentation will be offered to all councils and study participants. National Institute for Health and Care Research networks will be used to inform professionals and members of the public of the results, as well as local and national networks and key stakeholders. Findings will also be incorporated into undergraduate and postgraduate teaching programmes at the authors’ institutions.

Provenance and peer review: Not commissioned; externally peer reviewed.
