# Supplementary material for: Effectiveness of an intervention for reducing sitting time and improving health in office workers: three arm cluster randomised controlled trial
Source: BMJ. 2022 Aug 17;378:e069288. doi: 10.1136/bmj-2021-069288 (PMC9382450; doi:10.1136/bmj-2021-069288)
Supplement: Supplementary file 1 — Supplementary information: additional tables 1-8 [file edwc069288.ww.pdf]

Supplementary Table 1. Table outlining the changes to the study during its conduct

Change to the study	Details of the change	Date of change
Additional council recruited	Due to a lower than anticipated recruitment at the councils in the Greater Manchester area, Liverpool council was recruited.	June 2018
Sample size	When preparing the grant application, we visited our target councils and gathered details on the number of offices and people within these offices to inform our sample size calculation. However, at the start of recruitment, the observed average size and variability of cluster sizes were different to those assumed in the original sample size calculation due to a smaller number of participants within some offices expressing an interest in taking part. With the DMEC's guidance, the sample size was recalculated to ensure the study was adequately powered. Changing the average cluster size from 20 to 10, the variability in cluster size from 0.54 to 1.42 (cluster size range of 4-38), the inflation for loss to follow-up and non-compliance with the primary outcome from 30% to 40%, while keeping all other assumptions the same, required 690 participants from 72 clusters.	
Data collection time points	The 24-month data collection was due to start in May 2020. The impact of the COVID-19 pandemic on the ability to conduct the 24-month data collection was discussed at the trial DMEC and TSC. Further discussions were then held with the study funder. As a result, the decision was taken not to conduct 24-month follow-up. Therefore, daily sitting time at 12 months became the primary objective (not secondary) and the secondary objectives were not assessed at 24 months: the follow-up time points were now 3 and 12 months, rather than 3, 12, and 24 months	April-May 2020
Additional outcome	Reporting activPAL and Axivity data on workdays and non-workdays was not a pre-specified outcome and was added to the Statistical Analysis Plan	May 2020
Additional outcome	A clustered cardiometabolic risk score was not a pre-specified outcome and was added to the Statistical Analysis Plan	July 2020

Supplementary Table 2. Sensitivity analyses for changes in ***daily sitting time*** (min/day) at 12-month follow up (primary outcome) between participants randomised to standard care or intervention groups (SWAL and SWAL + Desk).

	Number of clusters (participants)			Baseline			Mean change from baseline to 12-month follow-up (SD)			Adjusted mean difference at follow-up (95% CI) | P-value		
	**Control**	**SWAL**	**SWAL+ Desk**	**Control**	**SWAL**	**SWAL+ Desk**	**Control**	**SWAL**	**SWAL+ Desk**	**SWAL vs. Control**	**SWAL+ Desk vs. Control**	**SWAL+ Desk vs. SWAL**
Intention to treat [a]	26 (267)	27 (249)	25 (240)	601.1 (83.6)	605.1 (84.4)	608.2 (79.0)	13.3 (74.8)	-10.1 (82.8)	-53.1 (81.6)	**-20.2 (-35.8 to -4.5) | 0.012**	**-63.1 (-78.4 to -47.7) | <0.001**	**-42.9 (-58.5 to -27.3) | <0.001**
Per protocol [a]	26 (164)	22 (158)	25 (167)	604.7 (76.0)	609.6 (75.1)	618.7 (72.1)	11.4 (73.5)	-12.9 (81.4)	-56.3 (77.0)	**-20.8 (-36.3 to -5.4) | 0.008**	**-61.2 (-76.5 to -45.9) | <0.001**	**-41.0 (-57.2 to -24.8) | <0.001**
Standardised to waking day (16hrs) [c]	26 (183)	27 (177)	25 (187)	453.2 (58.9)	460.0 (58.7)	461.6 (58.2)	9.6 (46.2)	-1.7 (53.8)	-30.2 (55.4)	**-10.3 (-20.4 to -0.3) | 0.04**	**-38.6 (-48.5 to -28.6) | <0.001**	**-28.3 (-39.0 to -17.6) | <0.001[e]**
Effect of number of valid activPAL days (complete-case):												
≥ 4 days [d]	26 (174)	27 (169)	25 (168)	595.8 (83.6)	603.0 (80.6)	608.7 (80.5)	15.1 (71.9)	-10.9 (80.2)	-50.6 (78.7)	**-22.1**	**-60.6**	**-38.8**

										**(-37.0 to - 7.1) | 0.004**	**(-75.6 to - 45.6) | <0.001**	**(-54.1 to - 23.5) | < 0.001**
≥ 1 workday [d]	26 (176)	26 (165)	25 (177)	596.5 (84.3)	604.0 (79.8)	610.4 (80.2)	15.0 (75.3)	-11.4 (79.8)	-54.2 (79.1)	**-22.4 (-37.3 to - 7.5) | 0.003**	**-63.2 (-77.8 to 48.6) | <0.001**	**-40.8 (-55.9 to - 25.8) | <0.001 [g]**
≥ 3 workdays [d]	26 (156)	26 (144)	25 (143)	597.1 (83.9)	605.0 (80.6)	612.0 (80.5)	16.5 (72.9)	-8.9 (79.7)	-54.7 (79.0)	**-21.5 (-37.4 to - 5.5) | 0.008**	**-65.1 (-81.1 to - 49.2) | <0.001**	**-43.9 (-60.6 to - 27.2) | <0.001 [h]**

[a] ≥1 valid day at baseline and 12 months. Adjusted for average daily sitting time at baseline, average waking wear time across baseline and 12 months, stratification factors area (Leicester; Salford; Liverpool) and cluster size category (Small <10; Large ≥10); [b] 97.5% confidence interval; [c] ≥1 day at baseline and 12 months. Adjusted for average daily standardized sitting time at baseline, stratification factors area (Leicester; Salford; Liverpool) and cluster size category (Small <10; Large ≥10); [d] Adjusted for average daily sitting time at baseline, average waking wear time across baseline and 12 months, and the stratification factors area (Leicester; Salford; Liverpool) and cluster size category (Small <10; Large ≥10).

Supplementary Table 3. Adjusted differences in average sitting time (mins/day) at 12 months for interactions between age and the intervention groups

	**Adjusted mean difference at follow-up (95% CI) | P-value**		
	**Average daily sitting time per year of age (for the control group)**	**Interaction with SWAL group**	**Interaction with SWAL + Desk group**
Daily sitting time	0.52 (-0.43 to 1.48) | 0.28	-0.38 (-1.83 to 1.08) | 0.61	-1.11 (-2.52 to 0.29) | 0.12
Daily sitting time during work hours	-0.12 (-1.03 to 0.80) | 0.80	-0.06 (-1.44 to 1.32) | 0.94	**-1.50 (-2.81 to -0.19) | 0.03**

Supplementary Table 4. Adjusted differences in average sitting time (mins/day) at 12 months for interactions between BMI and the intervention groups

	**Adjusted mean difference at follow-up (95% CI) | P-value**		
	**Average daily sitting time per unit increase in BMI (for the control group)**	**Interaction with SWAL group**	**Interaction with SWAL + Desk group**
Daily sitting time	1.09 (-0.76 to 2.93) | 0.25	-0.02 (-2.61 to 2.57) | 0.99	1.61 (-0.97 to 4.12) | 0.22
Daily sitting time during work hours	-1.50 (-3.24 to 0.24) | 0.09	0.43 (-2.08 to 2.93) | 0.74	**2.70 (0.26 to 5.13) | 0.03**

Supplementary Table 5. Sensitivity analyses for changes in ***sitting time during work hours*** (min/day) at 12-month follow up (primary outcome) between participants randomised to standard care or intervention groups (SWAL and SWAL + Desk).

	**Number of clusters (participants)**			**Baseline**			**Mean change from baseline to 12-month follow-up (SD)**			**Adjusted mean difference at follow-up (95% CI) | P-value**		
	**Control**	**SWAL**	**SWAL+ Desk**	**Control**	**SWAL**	**SWAL+ Desk**	**Control**	**SWAL**	**SWAL+ Desk**	**SWAL vs. Control**	**SWAL+ Desk vs. Control**	**SWAL+ Desk vs. SWAL**
Intention to treat [a]	26 (267)	27 (249)	25 (240)	359.1 (65.7)	355.0 (71.2)	358.3 (68.6)	-0.3 (60.9)	-10.3 (73.6)	-53.4 (85.4)	-11.5 (-26.5 to 3.5) | 0.132	**-54.0 (-69.0 to -39.0) | <0.001**	N/A.
Per protocol [a]	26 (157)	22 (149)	25 (157)	360.7 (59.5)	364.1 (65.4)	365.3 (56.7)	-0.1 (58.2)	-14.5 (71.3)	-59.5 (83.5)	-12.5 (-28.4 to 3.5) | 0.125	**-57.7 (-73.3 to -42.1) | <0.001**	N/A.
Standardised to working day (8hrs) [b]	26 (176)	26 (167)	25 (177)	352.3 (57.3)	352.7 (58.0)	356.2 (56.3)	3.3 (53.1)	-12.4 (59.8)	-59.9 (73.4)	**-14.9 (-28.6 to -1.2) | 0.033**	**-61.4 (-74.9 to -47.9) | <0.001**	**-46.6 (-61.0 to -32.1) | <0.001**
Effect of number of valid activPAL days (complete-case):												
≥ 3 workdays [c]	26 (156)	26 (144)	25 (143)	356.1	355.4	363.9	2.6	-10.6	-59.8	-12.5	**-60.3**	N/A.

				(67.2)	(71.3)	(61.5)	(61.7)	(69.4)	(85.3)	(-28.6 to 3.6)<br><br>| 0.127	**(-76.3 to -44.2)**<br><br>**| <0.001**	

[a] ≥1 valid day during work hours at baseline and 12 months. Adjusted for average sitting time during work hours at baseline, average wear time during work hours across baseline and 12 months, stratification factors: area (Leicester; Salford; Liverpool); cluster size category (Small <10; Large ≥10); [b]≥1 valid day during work hours at baseline and 12 months. Adjusted for average standardized sitting time during work hours at baseline, stratification factors area (Leicester; Salford; Liverpool) and cluster size category (Small <10; Large ≥10);[c] Adjusted for average sitting time during work hours at baseline, average wear time during work hours across baseline and 12 months; stratification factors: area (Leicester; Salford; Liverpool); cluster size category (Small <10; Large ≥10). N/A: test not conducted as one intervention group vs control was not significant.

Supplementary Table 6. Other activPAL secondary outcomes at baseline and change at 3 and 12 months compared to baseline

	Baseline value			Change at 3 months			Change at 12 months		
	**Control**	**SWAL**	**SWAL+Desk**	**Control**	**SWAL**	**SWAL+Desk**	**Control**	**SWAL**	**SWAL+Desk**
**Daily**									
MVPA Stepping (min/day)	27.1 (17.4)	27.0 (18.8)	26.5 (17.5)	-1.75 (12.9)	-0.45 (14.5)	-1.04 (12.6)	-2.23 (12.8)	-0.77 (14.5)	-0.22 (15.0)
Number of steps (steps/day)	9291.0 (3209.1)	9286.4 (3121.3)	9230.9 (3228.7)	-375.2 (2114.7)	-8.62 (2516.3)	-189.8 (2241.8)	-481.9 (2013.4)	-118.9 (2650.6)	-124.4 (2608.9)
Number of sit to upright transition (number/day)	53.7 (13.8)	53.6 (13.5)	52.3 (14.1)	-0.94 (9.02)	-0.35 (10.2)	-3.64 (9.92)	-0.86 (9.37)	-0.55 (9.95)	-3.43 (8.60)
**Working hours**									
MVPA Stepping (min/day)	13.0 (8.24)	12.5 (8.42)	12.8 (9.51)	-0.20 (7.68)	0.68 (8.40)	0.01 (8.61)	-0.54 (11.8)	0.36 (8.86)	1.43 (14.8)
Number of steps (steps/day)	3822.7 (1452.2)	3885.7 (1434.3)	3835.3 (1686.2)	-11.3 (1421.0)	194.9 (1501.2)	157.0 (1508.7)	-123.7 (1415.1)	202.3 (1448.3)	308.6 (1879.9)
Number of sit to upright transition (number/day)	27.5 (10.4)	28.2 (11.0)	25.8 (9.48)	0.27 (8.19)	0.57 (7.94)	-2.41 (8.37)	-1.60 (8.27)	0.26 (9.40)	-1.29 (9.45)
**Workdays**									
MVPA Stepping (min/day)	30.2 (19.0)	28.9 (20.8)	29.2 (20.0)	-1.62 (13.0)	0.26 (14.7)	-0.50 (13.9)	-2.03 (15.5)	0.22 (16.0)	0.01 (17.6)
Number of steps (steps/day)	9359.2 (3241.9)	9202.8 (3133.4)	9293.4 (3453.9)	-411.9 (2042.7)	130.0 (2391.2)	-136.7 (2385.4)	-410.2 (2228.7)	52.1 (2645.1)	-35.7 (2886.6)

Number of sit to upright transition (number/day)	55.8 (14.4)	55.2 (14.9)	53.3 (14.3)	-0.62 (9.50)	1.19 (10.8)	-4.09 (11.5)	-1.24 (9.82)	-0.02 (12.1)	-3.43 (10.5)
**Non-workdays**									
MVPA Stepping (min/day)	20.1 (21.2)	23.8 (24.4)	20.4 (22.4)	0.72 (24.3)	-1.91 (26.9)	-0.09 (25.2)	-1.48 (22.5)	-2.57 (26.1)	1.84 (23.1)
Number of steps (steps/day)	9051.8 (4150.4)	9467.5 (4979.4)	8941.5 (4166.9)	-17.0 (3939.0)	-407.9 (4982.4)	-51.4 (4419.1)	-343.8 (3813.8)	-482.9 (5269.2)	78.5 (4046.5)
Number of sit to upright transition (number/day)	51.8 (16.8)	50.8 (15.8)	50.3 (17.2)	1.52 (36.1)	1.70 (34.1)	4.48 (62.1)	-0.62 (13.9)	-0.32 (13.4)	-2.36 (13.2)

Supplementary Table 7. Axivity data (secondary outcomes) at baseline and change at 3 and 12 months compared to baseline

	Baseline value			Change at 3 months			Change at 12 months		
	**Control**	**SWAL**	**SWAL+Desk**	**Control**	**SWAL**	**SWAL+Desk**	**Control**	**SWAL**	**SWAL+Desk**
**Daily**									
Light activity (min/day)	182.8 (42.8)	179.7 (49.2)	186.4 (58.9)	4.35 (46.0)	1.60 (51.0)	-2.99 (57.5)	1.73 (60.0)	-0.23 (53.9)	0.73 (63.7)
MVPA (min/day)	44.9 (24.3)	42.6 (24.5)	43.3 (23.9)	0.22 (18.6)	2.52 (24.8)	-0.02 (18.3)	1.62 (30.9)	2.41 (26.4)	1.05 (20.1)
Sleep duration (min/day)	456.8 (54.7)	457.5 (55.8)	448.0 (54.2)	8.54 (53.5)	-1.17 (49.7)	8.28 (53.7)	1.64 (51.6)	-3.56 (48.9)	9.50 (55.7)
Sleep efficiency (per day) [a]	0.89 (0.05)	0.88 (0.05)	0.89 (0.05)	-0.00 (0.05)	0.00 (0.04)	-0.01 (0.08)	0.00 (0.04)	0.00 (0.04)	-0.00 (0.05)
**Work hours**									
Light activity (min/day)	65.9 (20.8)	68.7 (26.2)	64.0 (26.6)	2.73 (27.6)	0.55 (21.5)	3.76 (24.5)	1.22 (23.8)	3.23 (24.9)	7.23 (28.7)
MVPA (min/day)	17.7 (11.0)	16.7 (9.6)	17.8 (11.4)	0.80 (9.61)	3.01 (10.1)	0.30 (9.69)	-0.12 (10.2)	2.10 (9.63)	1.12 (12.0)
**Workdays***									
Light activity (min/day)	174.8 (41.6)	173.0 (48.7)	174.2 (57.5)	0.95 (44.5)	1.91 (49.8)	-3.88 (50.0)	2.80 (58.4)	0.03 (58.0)	4.77 (64.6)
MVPA (min/day)	47.3 (25.0)	44.0 (24.1)	46.1 (25.7)	0.15 (17.8)	3.88 (23.6)	-0.16 (19.0)	0.87 (28.9)	3.91 (25.7)	0.65 (22.0)
Sleep duration (min/day)	452.5 (65.9)	457.8 (68.1)	445.6 (65.7)	-7.85 (69.7)	-11.7 (64.8)	-2.43 (71.4)	-8.46 (61.1)	-10.8 (65.2)	-5.01 (66.1)
Sleep efficiency (per day) [a]	0.89 (0.05)	0.88 (0.06)	0.89 (0.05)	-0.00 (0.04)	0.01 (0.05)	-0.01 (0.07)	0.00 (0.04)	0.00 (0.05)	0.00 (0.05)
**Non-workdays***									
Light activity (min/day)	197.7 (55.9)	188.7 (55.3)	204.3 (64.9)	1.24 (53.5)	0.44 (48.8)	-7.60 (65.5)	3.85 (74.2)	0.43 (51.6)	-10.44 (64.7)
MVPA (min/day)	39.4 (31.4)	40.6 (36.8)	38.2 (29.2)	0.89 (34.0)	-1.13 (39.7)	0.31 (32.8)	3.38 (48.4)	0.99 (41.9)	-0.22 (26.3)

Sleep duration (min/day)	462.6 (66.3)	462.5 (70.2)	456.6 (65.3)	26.1 (78.0)	10.4 (78.5)	21.2 (84.9)	20.6 (81.6)	9.3 (82.2)	28.5 (90.4)
Sleep efficiency (per day) [a]	0.88 (0.05)	0.88 (0.06)	0.89 (0.04)	0.00 (0.06)	-0.01 (0.05)	-0.00 (0.05)	0.01 (0.05)	0.00 (0.05)	-0.00 (0.07)

***Data split by workdays and non-workdays were not pre-specified outcomes.** [a] Sleep efficiency (0.00-1.00), higher value indicates better sleep efficiency

Supplementary Table 8. Number of participants available for analysis at baseline and change at 3 and 12 months for all outcome variables

	Baseline			Baseline and 3 months			Baseline and 12 months		
	**Control**	**SWAL**	**SWAL+Desk**	**Control**	**SWAL**	**SWAL+Desk**	**Control**	**SWAL**	**SWAL+Desk**
**ActivPAL**									
Daily variables	257	237	229	210	200	202	183	177	187
Working hours variables	243	226	218	186	175	176	176	167	177
Workdays variables	244	226	218	187	175	176	176	165	177
Non-workdays variables	236	219	203	170	166	162	160	152	156
**Axivity**									
Daily									
Physical activity variables	255	235	228	203	197	202	182	170	184
Sleep variables	245	226	218	187	175	178	166	158	161
Work hours variables	239	224	213	181	173	171	162	146	159
Workdays									
Physical activity variables	240	226	213	182	179	171	171	160	170
Sleep variables	215	188	188	152	139	133	139	126	133
Non-workdays									
Physical activity variables	253	225	221	189	180	189	171	159	167
Sleep variables	233	206	201	165	145	160	143	132	139

**Self-reported lifestyle behaviours**									
Sleep quality (self-reported)	250	233	226	171	192	187	173	159	182
Typical working week									
No. of workdays	248	232	223	168	191	181	170	160	179
No. of hours worked per day	246	232	220	167	189	178	167	160	175
% of day spend in office	245	231	220	164	188	177	165	159	176
% of day spent at desk	246	230	219	165	186	176	165	158	176
Self-reported behaviours									
Workday sitting (%)	248	232	218	169	188	172	169	158	175
Workday standing (%)	244	228	217	166	185	171	165	155	172
Workday walking (%)	248	232	218	169	188	172	169	158	174
Workday prolonged sitting (%)	240	232	218	163	186	174	164	150	173
Sitting whilst working (min/week)	220	214	198	144	171	149	145	139	147
No. sitting breaks per hour	245	230	221	167	189	176	169	153	176
Weekdays (h/weekday)									
Sitting for transport	212	205	197	134	159	148	130	132	143
Sitting for TV viewing	205	202	201	132	158	152	124	127	141
Sitting for computer use	196	188	189	121	140	138	117	114	128
Sitting other activities	155	137	139	76	86	78	71	61	63

All sitting	144	133	129	71	79	68	65	57	57
Weekends (h/weekend day)									
Sitting for transport	211	193	194	131	142	144	128	119	139
Sitting for TV viewing	204	196	191	126	149	140	126	125	130
Sitting for computer use	192	189	184	115	135	126	113	116	116
Sitting other activities	154	136	137	75	86	79	73	64	62
All sitting	144	127	127	68	79	67	66	57	54
Weekly (h/day)									
Sitting for transport	203	188	190	127	138	139	123	117	131
Sitting for TV viewing	198	192	190	122	144	138	121	121	127
Sitting for computer use	186	182	179	110	128	124	109	110	110
Sitting other activities	147	131	130	70	81	72	68	57	55
All sitting	138	123	120	63	74	61	61	52	48
**Self-reported dietary behaviours**									
(≥1/day) Snack frequency	247	228	222	169	185	183	166	154	176
Soft drink consumption	247	230	225	168	187	187	169	155	180
Fruit consumption	250	230	223	171	188	183	171	155	177
Vegetable consumption	250	229	223	171	187	182	170	154	177

Alcohol intake									
Total units/week	166	153	145	102	115	101	96	88	97
**Physical Health**									
**Adiposity**									
Weight (kgs)	265	249	240	189	211	207	189	185	200
Waist circumference (cm)	266	248	240	188	209	207	190	182	196
Body fat (%)	264	246	238	187	206	203	188	181	194
BMI (kg/m^2)	265	249	240	189	211	207	189	185	200
**Blood pressure**									
Systolic BP (mmHg)	265	246	239	190	210	205	191	185	196
Diastolic BP (mmHg)	265	246	239	190	210	205	191	185	196
**Biochemical**									
HbA1c (mmol/mol)	260	245	233	176	189	184	187	175	183
HbA1c (%)	260	245	233	176	189	184	187	175	183
Total cholesterol (mmol/l)	263	245	235	188	202	199	187	179	191
HDL cholesterol (mmol/mol)	264	247	236	189	204	200	189	181	193
LDL cholesterol (mmol/mol)	260	241	227	177	193	186	178	168	174
Triglycerides (mmol/l)	264	247	236	189	204	200	189	181	193
Fasting glucose (mmol/l)	263	246	235	189	204	199	188	181	192
Cardiometabolic risk score	260	243	234	185	201	197	185	178	184
**Fatigue** [a]									

Physical	250	232	225	171	190	187	173	159	181
Mental	247	229	224	169	183	183	169	154	179
Global	247	229	224	171	188	186	172	156	180
**Musculoskeletal issues**									
**Issues in the last 3 months**									
Neck	241	230	220	163	187	182	166	152	171
Lower back	245	229	219	165	186	180	166	150	171
Upper extremity	247	231	223	168	187	184	170	156	178
Lower extremity	245	230	220	166	185	182	169	156	176
Any site	249	232	226	171	189	188	173	158	181
**Pain in the last 3 months**									
Neck	238	222	212	158	179	174	164	145	164
Lower back	245	228	217	165	185	177	166	149	169
Upper extremity	246	229	223	166	184	182	169	152	178
Lower extremity	244	229	220	162	183	181	168	153	175
Any site	249	232	226	171	188	187	173	157	180
**Psychological health**									
Anxiety	250	233	226	170	191	187	172	157	182
Depression	250	233	226	170	191	187	172	157	182
Stress	244	232	225	166	190	185	168	156	181
Wellbeing	244	231	226	165	189	186	168	157	181

Positive Affect	250	231	225	169	189	185	172	154	179
Negative Affect	250	230	225	170	188	185	173	153	179
Quality of Life									
Health utility score	249	231	223	169	189	184	171	156	178
Health state score	247	229	222	168	186	181	172	154	174
**Work-related outcomes**									
**Work engagement**									
Vigour	249	233	226	170	191	189	172	158	181
Dedication	249	233	226	170	191	189	172	158	181
Absorption	249	233	226	170	191	188	172	158	181
Overall	249	233	226	170	191	189	172	158	181
**Job performance**	248	233	225	169	190	187	172	157	180
**Job satisfaction**	249	233	225	170	190	187	172	157	180
**Occupational fatigue recovery**	248	233	226	170	192	189	172	161	181
**Workload and relations**									
Demands	249	232	224	170	191	184	173	160	181
Control	249	232	225	170	191	185	173	160	182
Support	249	232	225	170	191	185	173	159	182
**Organisational social norms**	246	230	226	168	190	188	171	159	182
**Social community**	246	230	226	167	190	188	171	159	181

**Support**									
Organisation	226	215	206	140	173	167	157	142	164
Manager	225	215	207	139	174	168	156	143	164
Colleagues	224	215	208	139	173	168	156	143	164
Family	220	215	205	136	174	162	152	142	161
**Work limitations**									
Time management	243	224	218	162	182	176	162	152	166
Physical demands	235	207	208	153	164	165	155	138	159
Mental-interpersonal demands	241	230	222	162	188	181	163	157	177
Output demands	244	230	222	164	185	177	167	156	175
Overall	249	232	224	170	191	184	173	159	180
**Absenteeism (self-report)**	226	219	216	N/A	N/A	N/A	149	139	162
**Absenteeism (records)**									
Episodes	202	193	203	N/A	N/A	N/A	202	193	203
Duration	202	193	203	N/A	N/A	N/A	202	193	203
